# Potential Toxic Elements in Farm Soils and Vegetables of Northern Bangladesh: Impact on Soil Health and Human Safety

**DOI:** 10.3390/jox16040127

**Published:** 2026-07-10

**Authors:** Aninda Sarker, Supti Mallick, Minhaj Uddin, Ronzon Chandra Das, Md. Harun Rashid, Md. Shohidul Alam, Quazi Forhad Quadir, Md. Zakir Hossen

**Affiliations:** 1 Laboratory of Plant Nutrition and Environmental Chemistry, Department of Agricultural Chemistry, Faculty of Agriculture, Bangladesh Agricultural University, Mymensingh 2202, Bangladesh; aninda.1902286@bau.edu.bd (A.S.); supti.ac@bau.edu.bd (S.M.); minhaj.24220920@bau.edu.bd (M.U.); ronzonbjri2020@gmail.com (R.C.D.); shohidul.alam@bau.edu.bd (M.S.A.); qfq@bau.edu.bd (Q.F.Q.); 2 Department of Agronomy, Faculty of Agriculture, Bangladesh Agricultural University, Mymensingh 2202, Bangladesh; mhrashid@bau.edu.bd

**Keywords:** soil contamination, metal accumulation, upper tolerable threshold, carcinogenic and non-carcinogenic risks, redundancy analysis (RDA)

## Abstract

Intensive vegetable production can increase the transfer of persistent toxic trace elements from agricultural soils into the food chain, particularly where agrochemical use, irrigation inputs, and local geochemical conditions are insufficiently characterized. This study was undertaken to assess toxic trace-metal contamination levels in soils and vegetables from two renowned vegetable-producing subdistricts—Shibganj and Kahaloo—in the Bogra district, Bangladesh. The study also estimated potential human health risks by evaluating the dietary intake of these elements. It measured Pb, Ni, Cd, and Cr content in six vegetables and their respective farm soils using an atomic absorption spectrophotometer (AAS). The average concentrations of Pb, Ni, Cd, and Cr in farm soils of Shibganj and Kahaloo subdistricts were 158.3 ± 8.83, 31.5 ± 5.25, 0.43 ± 0.08, and 14.1 ± 2.16 µg g^−1^ and 164.1 ± 4.60, 35.7 ± 6.91, 0.53 ± 0.14, and 9.37 ± 2.87 µg g^−1^, respectively. Soils collected from all locations in both subdistricts of Bogra fall under ‘moderate’ ecological risk. Regarding the pollution load index (PLI), 66.7% of Shibganj and 75.0% of Kahaloo sampling sites had a PLI > 1.0, confirming that ‘metal pollution exists.’ Based on the calculated bioconcentration factors (BCFs), Cr and Cd show a high tendency to migrate from soil to various vegetables in the study area, though the mean Cd BCF for brinjal in Shibganj exceeded 1.0 due to a single high observation. The results demonstrated that the edible parts of potatoes, onions, and chilies accumulate significant amounts of toxic trace elements. The calculated mean daily intake of Pb and Cr in all vegetables ranged from 0.33 to 1.21 mg person^−1^ day^−1^ and from 0.10 to 0.64 mg person^−1^ day^−1^, respectively, exceeding the upper tolerable intake limits. Similarly, dietary intake of potatoes showed both non-carcinogenic and carcinogenic risks, while brinjal showed only carcinogenic risks for adults. Redundancy analysis (RDA) indicates that the measured soil parameters are strong predictors of the response variables (trace element content in various vegetables). Overall, the results identified Pb-dominated soil contamination and human exposure to Pb and Cr associated with vegetables as the principal concerns. To address these issues, priority actions should be given to source apportionment and testing of various agricultural inputs. Additionally, before implementing site-specific remediation or issuing consumption advisories, these risks should be validated through metal speciation and bioaccessibility analyses.

## 1. Introduction

Vegetables are essential components of the Bangladeshi diet because they provide nutrients, vitamins, minerals, dietary fiber, and bioactive compounds. Their nutritional value, affordability, and year-round availability make them especially important for low- and middle-income households [1,2,3]. However, vegetables can also act as a pathway by which various contaminants, including toxic metals accumulated in agricultural soils, enter the human food chain. It is therefore essential that food-quality management and potential trace element uptake are actively evaluated alongside intensive agricultural cultivation.

The global rise in industrial and agricultural activities means metals and metalloids are present in the environment, raising ecotoxicological concerns. Non-essential toxic metals—cadmium (Cd), lead (Pb), nickel (Ni), chromium (Cr), mercury (Hg), and arsenic (As)—are dense and persistent, leading to their accumulation in agricultural soils, which is a major issue. Human activities such as industrial development, applying sewage sludge, using metal-contaminated agrochemicals, poor waste disposal, irrigating with wastewater, and discharging untreated effluents add trace metals to soils and vegetables [4,5,6,7,8,9]. Plants do not need toxic trace metals, but often absorb them in harmful amounts. Human exposure to toxic metals can occur through several pathways, including vegetables, fruits, cereals and other foods, drinking water, inhaled dust, and dermal contact; the relative contribution of each pathway varies with local diet and environmental conditions. However, a previous regional report assigned 90% of human exposure to toxic metals through the consumption of fruits and vegetables, with the remaining 10% from dust inhalation and skin absorption [10]. Toxic trace metals endanger human health, especially when present in amounts above the required levels. Even at low levels, these metals are dangerous to health because the body cannot excrete them efficiently [11,12]. Exposure can cause hepatic and renal dysfunction, intellectual incapacity, cerebrospinal disruption, diarrhea, hookworm infection, and corticobasal degeneration in the brain and liver [13,14,15]. Furthermore, Brown [16] stated that human exposure to toxic metals is a significant risk factor for cancer. Hence, to assess health risks, it is vital to measure levels of toxic metals in widely consumed local fruits and vegetables [10].

Bogra is a key agricultural center in Bangladesh. It produces vegetables at high intensity and supports the country’s food security. Cropping intensity in Bogra often exceeds the national average [17,18]. However, industrial activities and urbanization bring the potential for toxic metal contamination in farmland [19,20]. Additionally, farmers routinely use excessive fertilizers and pesticides contaminated with toxic metals [4,7]. Despite these risks, there is a lack of detailed data on the extent of metal contamination in Bogra soils and on the accumulation of these metals in locally grown vegetables and crops. Specifically, few studies have systematically investigated long-term accumulation and toxicity of metals in the vegetable–soil system in high-intensity farming regions like Bogra, nor have they clearly established how anthropogenic activities contribute to trace-metal contamination in farmland. This limited knowledge represents a significant research gap and hinders effective management. Furthermore, the monitoring of toxic elements in farmland and agricultural produce is not well implemented, not only in Bangladesh but also in many other countries worldwide. As a result, Bogra, like other growing urban areas, faces challenges with environmental quality and chemical exposure. Hence, this study specifically aims to (i) quantify toxic trace metal contamination in soils and vegetables from Bogra, (ii) compare estimated dietary intake of these metals against the upper tolerable intake limit (UTIL), and (iii) assess human health risks resulting from consumption of vegetables grown in Bogra.

## 2. Materials and Methods

### 2.1. Soil and Vegetable Sampling

**Figure 1:**
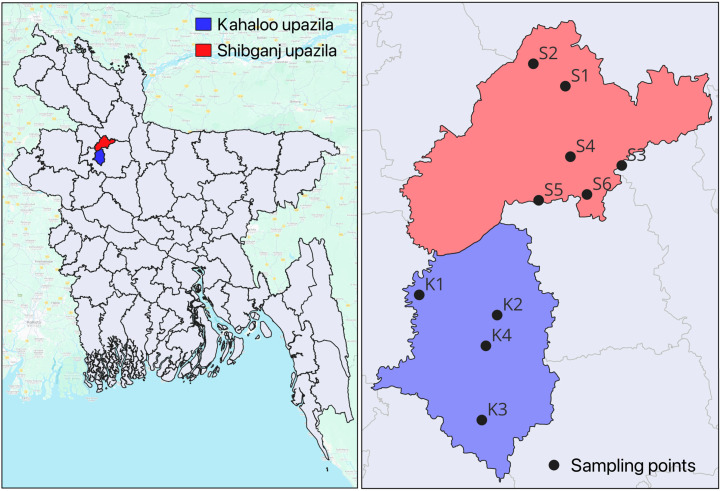
Map showing the sampling locations for soil and various vegetables collected from Kahaloo (blue/K) and Shibganj (red/S) subdistricts in the Bogra district, Bangladesh.

Sampling was carried out from December 2024 to January 2025 in two subdistricts of the Bogra district: Shibganj and Kahaloo. A total of six commonly consumed vegetables, namely brinjal, country bean, potato, radish, onion, and chili, were gathered in triplicate from six and four farms situated in Shibganj and Kahaloo, respectively (Figure 1). During collection, sample homogeneity in terms of type, size, and color was maintained strictly. Regarding vegetable consumption, brinjal, country bean, and potato are consumed cooked, whereas radish, onion, and chili are consumed both cooked and raw (as salads). Furthermore, onions and chili are also utilized as culinary seasonings or spices. However, farms were selected based on the availability of the six vegetables listed above across all locations. Soil samples were also collected from the same farmstead in triplicate using a soil auger and subsequently placed into nylon bags for analysis. Hence, a total of 180 (6 × 10 × 3) vegetable and 30 (10 × 3) soil samples were collected for this investigation. The protocol outlined by Singh et al. [21] was used to conduct both soil and vegetable sampling.

### 2.2. Processing of Soil and Vegetable Samples

After collecting both soil and vegetable samples, they were brought to the Laboratory of Plant Nutrition and Environmental Chemistry, Department of Agricultural Chemistry, Bangladesh Agricultural University, where they were processed and prepared for chemical analysis. First, all vegetable samples were meticulously washed with tap water and then with deionized water. Subsequently, the edible portions were cut into small pieces for dehydration. The vegetable and soil samples were air-dried, then oven-dried at 50 °C until a consistent weight was achieved. Finally, the samples were pulverized, sieved, and stored in sealed plastic bags.

### 2.3. Vegetable and Soil Samples Digestion

To determine the concentrations of toxic trace elements in the vegetable samples, the specimens were digested using the wet oxidation method. Precisely 1.0 g of a powdered, oven-dried vegetable sample was placed in a 150 mL conical flask. The flask contained 10 mL of an acid mixture (HNO_3_:HClO_4_ = 2:1). Digestion occurred in a sand bath at approximately 180–200 °C. Heating continued until white vapors appeared and the digest became colorless. After cooling, the contents were diluted with deionized water. The mixture was filtered into a 100 mL volumetric flask using Whatman No. 42 filter paper. The volume was made up to the mark with deionized water. Before further analysis, the extract was stored in labeled, airtight containers and kept refrigerated. All samples were digested in three independent duplicates. Soil samples were collected in 15 mL Teflon (PTFE) vials to determine total trace metal concentrations. The methodology described by Tessier et al. [22] was followed in this study, with minor adjustments detailed by Zakir and Shikazono [23].

### 2.4. Estimation of Soil Properties and Trace Metals

This study estimates soil physicochemical properties—pH, electrical conductivity (EC), organic carbon (OC), organic matter (OM), and textural classes—using techniques outlined by Singh et al. [21]. Subsequently, the concentrations of Pb, Cd, Ni, and Cr in soil and vegetable extracts were measured using an atomic absorption spectrophotometer (AAS) (SHIMADZU, AA-7000, Tokyo, Japan). Calibration for all trace elements was performed according to the manufacturer’s guidelines, establishing a minimum detection limit of 0.01 to 0.005 μg g^−1^. Comprehensive calibration data for the AAS during operation are shown in Table S1.

### 2.5. Quality Assurance and Quality Control (QA/QC)

Standard operating procedures and QA/QC protocols ensured data accuracy. To avoid cross-contamination, glassware and equipment were first acid-washed and then rinsed with deionized water. Duplicates (repeated samples), rinse blanks (clean water checked for contamination), and a full chain of custody (a record of sample handling) were maintained for quality checks. In addition, before each batch, blanks (samples without analyte) and matrix spikes (samples with known analytes) were used to verify recovery and accuracy. Reproducibility was calculated at less than 10%, further confirming data accuracy. Moreover, a certified reference material (CRM), JLk-1 (lake sediment), was included in this study to test the analytical methods; concentrations of trace metals in CRM aqueous solutions were assessed using the same methodology, and recoveries ranged from 91.9% to 102.8% (Table S2).

### 2.6. Soil Pollution Assessment

The main indices used to assess trace metal pollution in the soils of vegetable-cultivating areas in the Bogra district are the contamination factor (CF), pollution load index (PLI), and potential ecological risk index (PERI). These indices indicate soil contamination levels in the study area.

#### 2.6.1. Contamination Factor

Barbieri [24] states that CF is calculated by dividing the concentration of a trace metal in a soil/sediment sample by its benchmark value. In this study, the mean shale value from Turekian and Wedepohl [25] serves as the benchmark. CF values are categorized as follows: CF < 1.0 (low contamination), 1.0 ≤ CF < 3.0 (moderate), 3.0 ≤ CF < 6.0 (considerable), and CF > 6.0 (very high) [24,26].

#### 2.6.2. Pollution Load Index

The PLI assesses the area’s overall pollution level. It also measures the cumulative negative effects at a location. Tomlinson et al. [27] described the computation method for PLI, which is used here.

(1) $$PLI=[{CF}_{1}\times {CF}_{2}\times {CF}_{3}\times \ldots \times {{CF}_{n}]}^{1/n}$$

Here, n is the number of trace metals assessed, and CF_n_ is the concentration factor of the nth trace metal. The three PLI levels are: PLI < 1.0 (no metal pollution exists), PLI = 1.0 (only baseline pollution), and PLI > 1.0 (metal pollution exists) [27].

#### 2.6.3. Potential Ecological Risk Index

Hakanson [28] designed a system for assessing ecological risks in soil and sediment pollution management. The system’s risk is calculated by summing the single potential ecological risk factors (SPERFs). SPERF is measured by multiplying the toxicity coefficient (TC) of a particular trace metal by the contamination factor (CF). The designated TC values were 2 for Cr, 5 for both Pb and Ni, and 30 for Cd [26,28,29]. According to Hakanson [28], there are five categories of SPERF: SPERF < 40 (low); 40 ≤ SPERF < 80 (moderate); 80 ≤ SPERF < 160 (considerable); 160 ≤ SPERF < 320 (high); and SPERF ≥ 320 (very high). As regards PERI, there are four classes: PERI < 95 (low); 95 ≤ PERI < 190 (moderate); 190 ≤ PERI < 380 (considerable); and PERI ≥ 380 (high) [26,28].

### 2.7. Health Risk Assessment

#### 2.7.1. Trace Metal Intake Rate

Trace metal intake rate was calculated as outlined by Zakir et al. [30], then compared with the upper tolerable intake limits (UTILs) for each toxic trace element.

(2) $$Trace\;metal\;intake\;\left(mg\;{person}^{-1}\;{day}^{-1}\right)=\frac{AIRV\times c}{1000}$$

Here, *AIRV* means the average intake rate of vegetables (g person^−1^ day^−1^). The variable c denotes the calculated mean of toxic elements in vegetable samples (μg g^−1^, fresh weight). According to HIES [31], the daily mean vegetable consumption rate for people in Bangladesh is 167.3 g person^−1^ day^−1^.

#### 2.7.2. Chronic Daily Intake (CDI)

The USEPA’s exposure procedure [32] was employed to evaluate cancer and non-cancer risks by computing chronic daily intakes (mg kg^−1^ day^−1^) of toxic trace elements from the daily oral consumption of different vegetables.

(3) $$CDI\;\left(mg\;{\mathrm{kg}}^{-1}\;{\mathrm{day}}^{-1}\right)=\frac{\left(VIR\times C_{Vegetable}\times EF\times ED\right)}{BW\times AT}$$

Here, *VIR* means the vegetable intake rate, and *C_Vegetable_* indicates the concentration of toxic metals in vegetables. According to BBS [33], the calculated daily mean consumption rates for radish, country bean, brinjal, potato, onion, and chili were 5.04, 1.65, 8.79, 40.0, 0.75, and 0.70 g person^−1^ day^−1^, respectively. EF is the exposure frequency (6 months for radish and country bean, and 12 months for other vegetables). ED means exposure duration (66.3 years for an adult, by deducting the childhood period of 6 years from the total life expectancy in Bangladesh). AT is the period of exposure for non-carcinogenic effects (365 × ED), and BW denotes the mean body weight (60 kg for an adult in Bangladesh) [26].

#### 2.7.3. Non-Carcinoma Health Risk

The non-carcinogenic risks for an adult from toxic trace elements in locally cultivated vegetables were assessed using hazard quotients (HQs), calculated by dividing each element’s measured CDI by its oral reference dose (RfD). RfDs for Pb, Cd, Cr, and Ni were 0.0036, 0.001, 0.001, and 0.02 mg kg^−1^day^−1^, respectively [9,26,34]. Total HQs for each vegetable were summed to produce a hazard index (HI) that indicates exposure to multiple potentially hazardous compounds. While individual HQs were below 1, their aggregate intake could still affect health if the HI exceeds 1.

#### 2.7.4. Carcinogenic Health Risk

The probability that residents at the study sites may develop cancer from consuming potentially toxic trace elements in local fresh vegetables was determined for an adult. Incremental lifetime cancer risk (ILCR) was calculated by multiplying the obtained CDI by the cancer slope factor (CSF). The CSF values for Pb, Ni, and Cd were 0.0085, 0.91, and 15.0 mg kg^−1^day^−1^, respectively [26,34,35]. Chromium was excluded due to the absence of a CSF value. For safety, the acceptable ILCR value ranges from 10^−6^ to 10^−4^ [32].

### 2.8. Statistical Analysis

The data was analyzed using the statistical software application “R” (4.4.1) [36]. Prior to the statistical analyses, the data’s homogeneity was examined using the Shapiro-Wilk method. Nonparametric Kruskal–Wallis tests were used for mean comparisons. Redundancy analysis (RDA) was performed to evaluate relationships between soil physicochemical properties and toxic element concentrations in vegetables grown at the study locations. Toxic metal concentrations in the vegetables were treated as response variables. Soil characteristics were considered explanatory variables. Because the trace element concentration data were right-skewed, they were log-transformed before constructing the RDA model. Soil variables were standardized with a z-score transformation. RDA was conducted using the ‘vegan’ package in R [37] and ‘R’ [36]. The overall significance of the RDA model and canonical axes was tested using Monte Carlo permutation tests. The ‘ggplot2’ package was used for biplot visualization. Since the ordination biplot was cluttered due to many variables, only those well represented in the two-dimensional space were retained for graphical representation. Variables were retained by estimating the squared cosine (cos^2^) for each variable on the first two canonical axes. Only variables with cos^2^ ≥ 0.35 were kept.

## 3. Results and Discussion

### 3.1. Soil Physicochemical Properties

**Figure 2:**
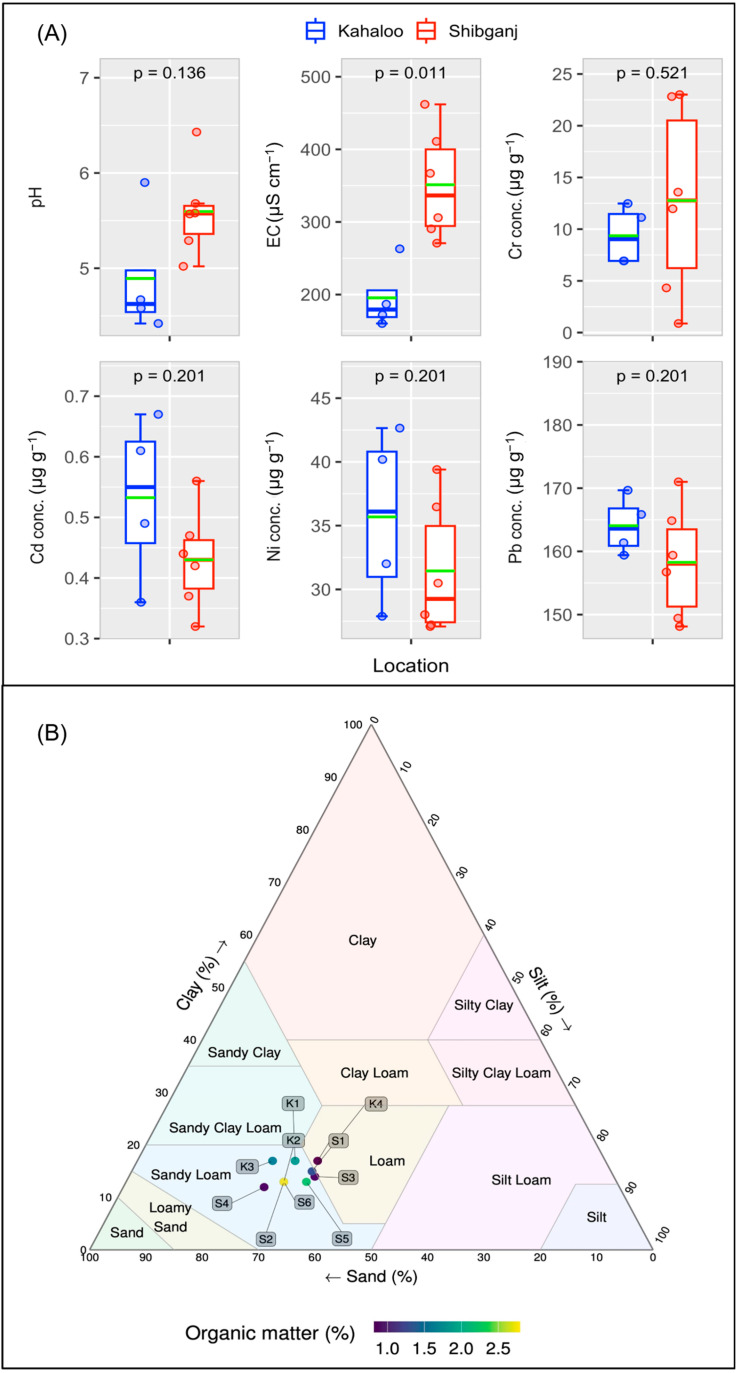
Physicochemical properties of soil. Figure (**A**) represents toxic trace metal contents in soils along with pH and EC. The lower and upper box boundaries are the 25th and 75th percentiles, respectively, whereas the colored horizontal line inside the box is the median, and the black line represents the mean value. The lower and upper error lines correspond to the 10th and 90th percentiles, respectively. Data points falling outside the 10th and 90th percentiles are the outliers. The *p*-values represent mean comparisons (nonparametric Kruskal–Wallis test) of the trace metal contents in soil collected from two sub-districts. Figure (**B**) shows soil textural classes and organic matter content in both the subdistricts (S = Shibganj & K = Kahaloo) of the Bogra district.

It is well established that agricultural soil quality is largely influenced by complex interactions among pH, electrical conductivity (EC), organic matter (OM), and texture. The physicochemical properties of vegetable-cultivating soils in farmers’ fields of Shibganj and Kahaloo subdistricts in the Bogra district are presented in Table 1 and Figure 2. The mean pH values in soils of Shibganj and Kahaloo were 5.60 ± 0.48 and 4.89 ± 0.68, respectively. This indicates that the soils of both locations were acidic. Although the numerical difference was 0.71 pH units, the Kruskal–Wallis test did not indicate statistical significance (*p* = 0.136; Figure 2); therefore, the result should be interpreted as a descriptive difference rather than evidence of a location effect. Similar observations for soils of Bogra were reported by Naher et al. [38]. However, no significant difference was observed between the subdistricts (Figure 2). The average EC value for Shibganj was 351.2 ± 75.2 µS cm^−1^, and for Kahaloo, it was 195.5 ± 46.3 µS cm^−1^. Soils with lower EC have low soluble salt concentrations, while higher EC indicates salinity problems. The results show that the soils in both subdistricts are classified as non-saline (EC < 2000 µS cm^−1^), indicating low salinity at every study site [39]. Arnold et al. [40] claim that soil EC is a useful indicator for agricultural management. The plant or plants to be produced must be considered when interpreting the EC of a given soil. Mean organic matter ranged from 0.82 ± 0.44% to 2.26 ± 0.52% in Shibganj and 1.25 ± 0.67% to 2.79 ± 0.64% in Kahaloo. According to Naher et al. [38], OM in Bogra soils ranged from 0.96% to 3.04%, consistent with these findings. The present results indicate that OM is lower in both subdistricts, particularly in Shibganj. Lower soil OM levels imply reduced nutrient availability, reduced microbial activity, and possible long-term deterioration, necessitating management adjustments to boost output [41]. Additionally, a number of trace elements were significantly positively correlated with soil organic matter [42]. The amount of metal available to plants is decreased when the soil contains more organic matter because trace elements become firmly bound to it and form metal chelate structures [42]. Soil texture is crucial because it influences many other soil processes and characteristics. These include soil strength, water movement, available water-holding capacity, ease of pollutant seepage into groundwater, and inherent fertility [43]. In Shibganj, soil texture was sandy loam to loam, with a mean sand content of 56.70 ± 4.46%, silt content of 29.67 ± 3.14%, and clay content of 13.63 ± 1.75%. In Kahaloo, soil textural classes were sandy clay loam, sandy loam, and loam, with average sand content of 56.54 ± 3.00%, silt content of 28.00 ± 3.27%, and clay content of 15.46 ± 1.91% (Figure 2B). The results show that all physicochemical parameters studied differed between the subdistricts. Although the variations were insignificant, these differences highlight the need for personalized soil management practices to enhance productivity. Understanding these soil properties is essential for optimizing crop growth and sustainable farming in the study area.

Toxic element contamination in agricultural farmland soils in Bangladesh is a serious problem. In this context, this study determines the concentration of Pb, Cd, Ni, and Cr in farmers’ field soils from the Shibganj and Kahaloo subdistricts of the Bogra district. The results obtained are shown in Table 1 and Figure 2. Specifically, the mean content of Pb, Cd, Ni, and Cr was 158.3 ± 8.83, 0.43 ± 0.08, 31.45 ± 5.25, and 14.08 ± 2.16 µg g^−1^, respectively, in soils of Shibganj, and 164.1 ± 4.60, 0.53 ± 0.14, 35.69 ± 6.91, and 9.37 ± 2.87 µg g^−1^, respectively, in soils of the Kahaloo subdistrict. Hence, it is apparent that the levels of Pb, Cd, and Ni were higher in Kahaloo soils than in Shibganj soils, suggesting greater contamination in that subdistrict. In contrast, Cr content was higher in Shibganj soils. Together, these findings highlight the variability in trace-element distribution across regions. Such variations may reflect distinct sources of pollution or soil characteristics that influence trace element deposition. The main factor influencing variance in trace element levels among different soils was changes in soil lithology. This observation is consistent with Taghipour et al. [44]. They reported that differences in lithology can explain the variability in trace element concentration, which may be highly varied in regions with differing parent material and soil conditions. Another important aspect observed is that the soils of both subdistricts are acidic. Acidic soils increase the mobility of trace metals, increase the risk of crop uptake, and contaminate the food chain [45]. Furthermore, the fraction of elements in question affects how different metallic substances dissolve. Specifically, in the acidic pH of the soil, the fractions associated with oxides, hydroxides, carbonates, or minerals are significantly more mobile [46]. A comparison of these findings with previous research reveals consistency across studies. For instance, Bushra et al. [34] reported Pb, Ni, Cd, and Cr levels in Jamalpur farmers’ field soils, ranging from 9.1–23.7, 14.1–25.1, trace-0.67, and 45.8–75.3 µg g^−1^, respectively. As we can see, the results from this investigation mirror those limits closely, except for Pb and Cr. Additionally, it should be noted that Samma et al. [47] reported 11.95 to 110.7 µg g^−1^ Cr in different farm soils of Bogra, which was comparatively higher than the present study results.

This section compares different concentrations of toxic trace metals in vegetable-cultivating soils from farmers’ fields in the Bogra district, Bangladesh, with various reference values, as outlined in Table 2. From Table 2, it is apparent that the present study’s average for Ni and Cr was lower than those of all the reference standard values listed. In contrast, the study’s mean Cd content was higher than the average shale value [25], the crustal average [48], and the upper continental crust value [49]. However, the Cd content was lower than the USEPA soil toxicity reference value [50], the Canadian soil quality guideline [51], and the Netherlands soil quality guideline [52]. Conversely, the Pb content was much higher than all the reference standard values. Majumder et al. [53] identified various industrial operations, sewage sludge, agrochemicals, organic manures, compost, and atmospheric deposition from mining and smelting as the main sources of Pb pollution in soils. Furthermore, Aktaruzzaman et al. [7] also found that phosphatic fertilizer and pesticide applications raised Pb levels in agricultural soils without industrial effluents. Together, this report highlights that even in the absence of industrial contamination, agricultural practices such as the application of phosphatic fertilizers and pesticides can remarkably contribute to Pb deposition in soils. Therefore, to reduce soil pollution, it underlines the need for careful management of agricultural inputs, particularly the use of high-quality agrochemicals.

### 3.2. Soil Pollution Status

**Figure 3:**
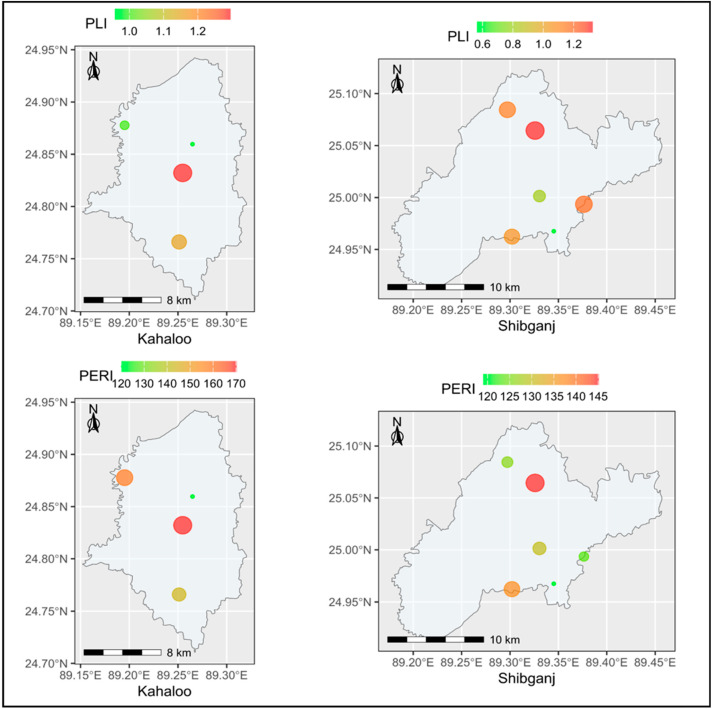
The bubble plot shows levels of toxic trace metal contamination in the soils of farmers’ fields in the Shibganj and Kahaloo subdistricts of the Bogra district, Bangladesh.

This study used CF, PLI, and PERI to systematically assess soil pollution in farmers’ fields in the Shibganj and Kahaloo subdistricts of the Bogra district. Summary statistics of CF, PLI, SPERF, and PERI for soils growing various vegetables in these areas are shown in Table 3. In Shibganj, calculated CF values for Cd ranged from 1.60 to 2.78, indicating ‘moderate’ contamination. In Kahaloo, CF values ranged from 1.78 to 3.36, marking ‘moderate’ contamination at half the sites and ‘considerable’ contamination at the other half [24] (Table S4). All locations in both subdistricts had ‘very high’ Pb contamination (CF > 6.0). However, CF values for Ni and Cr remained below 1.0 at all sampling sites, suggesting ‘low contamination’ (Table S4) and that these elements are not a concern. According to Shirani et al. [54] and Mishra and Lal [55], PLI helps measure contamination and metal pollution and guides further research or cleanup needs. Results showed that 66.7% of Shibganj and 75.0% of Kahaloo sampling sites had PLI > 1.0, confirming that ‘metal pollution exists’ and further study or remediation is needed. Other locations, with PLI < 1.0, indicated ‘no metal pollution exists’ [27]. Rahman et al. [56] reported that 42.0% soils in the wetlands of the Sylhet basin of Bangladesh showed high-level PLI values. However, Figure 3 illustrates the degree of potential trace metal contamination, helping identify affected areas. Overall, the study pinpointed locations with significant Pb and Cd contamination that warrant attention to prevent risks to crops and human health.

The calculated SPERF and PERI values for soils cultivating various vegetables in the Shibganj and Kahaloo subdistricts are presented in Table 3. Specifically, in Shibganj, SPERF values for Cd, Pb, Ni, and Cr ranged from 48.0 to 83.4 (mean 64.4), 59.3 to 68.4 (mean 63.3), 1.81 to 2.63 (mean 2.10), and 0.02 to 0.46 (mean 0.26), respectively. Similarly, in Kahaloo, SPERF values ranged from 53.4–100.8 for Cd (mean 79.9), 63.8–67.9 for Pb (mean 65.6), 1.86–2.85 for Ni (mean 2.38), and 0.14–0.25 for Cr (mean 0.19). All sites showed ‘moderate’ ecological risk due to Pb contamination (40 ≤ SPERF < 80) [28]. For Cd, 83.3% of Shibganj and 50.0% of Kahaloo sites had ‘moderate’ risk, while the remaining 16.7% in Shibganj and 50% in Kahaloo indicated ‘considerable’ risk. In contrast, SPERF values for Ni and Cr in both subdistricts consistently remained below 40.0, indicating ‘low contamination’ [28], so these elements are not a concern. Overall, the sequence of ecological risks based on SPERF values from least to greatest in both subdistricts was: Cr < Ni < Pb < Cd.

Weissmannová and Pavlovsk [57] note that the PERI index integrates toxicology, environmental chemistry, and ecology, assessing ecological risk from toxic trace elements. In Shibganj, PERI values ranged from 119.0 to 145.2, with a mean of 130.1; in Kahaloo, they ranged from 120.0 to 170.2, with a mean of 148.1 (Table 3). According to Hakanson’s [28] four PERI classes, all locations in both subdistricts fall under ‘moderate’ ecological risk (95 ≤ PERI < 190). These findings closely mirror the calculated values of PLI. However, Samma et al. [47] documented low ecological risk at six sites in the Bogra district. Figure 3 visually highlights sites with trace metal contamination based on PERI, thereby identifying the most affected areas. Overall, the calculated PERI enables the systematic identification of at-risk sites, supporting targeted interventions to mitigate ecological hazards.

### 3.3. Trace Element Contents in Vegetables

**Figure 4:**
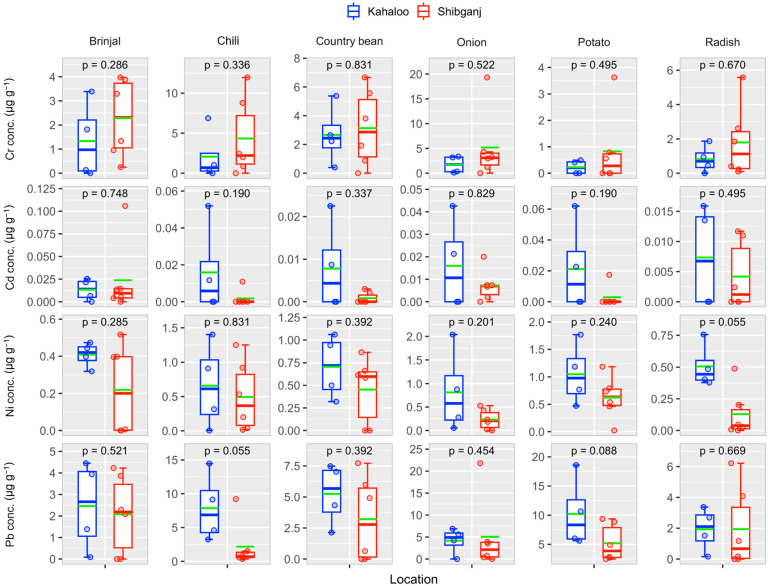
Trace element concentration in different vegetables collected from farmers′ fields in the Shibganj and Kahaloo subdistricts of the Bogra district. The lower and upper box boundaries represent the 25th and 75th percentiles, respectively. The colored horizontal line inside the box is the median, and the green line represents the mean value. The lower and upper error lines correspond to the 10th and 90th percentiles, respectively. Data points falling outside the 10th and 90th percentiles are the outliers. The *p*-values represent the mean comparison (nonparametric Kruskal–Wallis test) of trace metal contents in vegetables collected from the two subdistricts of the Bogra district.

This study focuses on measuring four toxic trace elements in six different vegetables cultivated across two subdistricts of Bogra, highlighting their concentrations and associated health risks. Figure 4 illustrates trace element concentrations in vegetables collected from different farmers’ fields in the Shibganj and Kahaloo subdistricts of the Bogra district. On the other hand, Table 4 presents the summary statistics of trace elements, their daily intake from various vegetables cultivated in the Bogra district of Bangladesh, and the upper tolerable daily intake limit (UTIL). In Bangladeshi cuisine, radish is a common winter vegetable. It is praised for its low price, strong flavor, and versatility in the kitchen, as it can be used in both cooked and raw salads. It is typically consumed for nearly half the year. The study estimated the amount of Pb, Ni, Cd, and Cr in radish grown in farmers’ fields of Bogra ranged from <0.005–6.22, <0.01–0.49, <0.005–0.012, and 0.10–5.58 µg g^−1^ in the Shibganj subdistrict and 0.16–3.38, 0.38–0.76, <0.005–0.016, and <0.01–1.87 µg g^−1^ in the Kahaloo subdistrict, respectively (Table 4). Biswas et al. [9] reported a comparatively lower amount of Cd and Pb in radish samples collected from Mymensingh City, Bangladesh. The study measured Pb and Ni in radish from all (12) samples of Kahaloo and Cr in all samples of Shibganj. Table 4 clearly shows that the mean daily intake of Pb and Cr from radish exceeds the UTIL, raising health concerns about these two elements, especially Cr, as its intake was nearly four times higher than the UTIL. Similar to the radish, the country bean is another vital winter legume in Bangladesh and offers a cheap and nutrient-dense source of protein, vitamins, and minerals for people of all income levels. It is also typically consumed for nearly half the year. The study quantified the concentrations of Pb, Ni, Cd, and Cr in country beans cultivated in farmers’ fields in Bogra, revealing ranges of <0.005–7.72, <0.01–0.86, <0.005–0.003, and <0.01–6.67 µg g^−1^ in the Shibganj subdistrict and 2.14–7.48, 0.32–1.06, <0.005–0.023, and 0.40–5.38 µg g^−1^ in the Kahaloo subdistrict, respectively (Table 4). However, Kharkwal et al. [58] reported comparatively lower amounts of Pb and Cd in cooked bean samples from Punjab, India. The present study quantified Pb and Ni in country beans from all twelve samples of Kahaloo, but their contents were below the detectable limit in 33.3% of the samples collected from the Shibganj subdistrict. Table 4 clearly indicates that the mean daily intake of Pb and Cr from the dietary intake of country bean was about 3 and 8 times above the UTIL, raising health concerns about human intake of these toxic elements. Overall, the results showed varying concentrations of trace elements in both winter vegetables. However, some samples exceeded the safe intake limit, particularly Pb and Cr. Hence, it is important to monitor and manage trace element contamination in cultivated crops in the farmlands of both subdistricts of Bogra.

Brinjal’s prominence as Bangladesh’s second most significant vegetable, after potatoes, underpins its importance in terms of both yield and cultivation area. Given its popularity and year-round availability, it is often called the “king of vegetables.” This study focuses on quantifying the concentrations of Pb, Ni, Cd, and Cr in brinjal from farmers’ fields in Bogra. Findings revealed that concentrations in the Shibganj subdistrict were <0.005–4.23, <0.01–0.52, <0.005–0.106, and 0.25–3.97 µg g^−1^, and in Kahaloo were 0.08–4.45, 0.32–0.47, <0.005–0.025, and <0.01–3.39 µg g^−1^, respectively (Table 4). All Kahaloo samples contained Pb and Ni, while all Shibganj samples had Cr. Cd was detected in 83.3% and 75.0% of samples from Shibganj and Kahaloo, respectively. Bushra et al. [34] found lower concentrations of these metals in brinjal from farmers’ fields in the Jamalpur district, while Biswas et al. [9] found slightly more Cd but less Pb in brinjal from the Mymensingh district marketplaces. Table 4 makes clear that the mean daily intake of Pb and Cr from brinjal was above the UTIL, posing human health concerns.

Onions and chilies, both essential to Bangladeshi cuisine, were analyzed in this study for trace element contamination and related health risks. Concentrations of Pb, Ni, Cd, and Cr in onions from farmers’ fields in Bogra ranged from <0.005–21.80, <0.01–0.53, <0.005–0.02, and <0.01–19.31 µg g^−1^ in Shibganj and <0.005–6.88, 0.05–2.04, <0.005–0.043, and 0.14–3.35 µg g^−1^ in Kahaloo (Table 4). All four elements were found in 83.3% of onion samples from Shibganj, while Kahaloo onions contained Ni and Cr in 100% of samples, Pb in 75%, and Cd in 50%. Ud Din et al. [59] observed similar Ni (0.89 µg g^−1^), higher Cd (0.61 µg g^−1^), but lower Cr (1.13 µg g^−1^) and Pb (3.04 µg g^−1^) in onions from Pakistan and Afghanistan. Gebreyohannes and Asgedom [60] reported 0.75 ± 0.05 µg g^−1^ for Pb, 0.41 ± 0.04 µg g^−1^ for Cd, and 1.98 ± 0.27 µg g^−1^ for Cr in onions grown in Ethiopia. However, Shokri et al. [61] reported 0.04, 0.14, and 0.10 µg g^−1^ Cd and 0.23, 0.20, and 0.24 µg g^−1^ Pb in yellow, red, and white varieties of onion in Iran. On the other hand, the measured concentrations of Pb, Ni, Cd, and Cr in chili cultivated in farmers’ fields in Bogra reveal ranges of 0.34–9.21, 0.02–1.25, <0.005–0.011, and <0.01–11.97 µg g^−1^ in Shibganj and 3.24–14.46, 0.01–1.40, <0.005–0.052, and <0.01–6.88 µg g^−1^ in Kahaloo (Table 4). Pb and Ni were detected in all chili samples from both subdistricts. Zhou and Liu [62] reported lower values for Cr (0.95 ± 0.30 µg g^−1^), Pb (0.89 ± 0.27 µg g^−1^), and Cd (0.063 ± 0.017 µg g^−1^) in dried chili from China. Table 4 shows that the mean daily intake of Pb and Cr from onions and chilies exceeded the UTIL, especially as these vegetables are often consumed raw. This finding indicates a potentially significant health risk for consumers, demonstrating the importance of monitoring and mitigating trace element contamination in these commonly eaten salad vegetables.

**Table 4:** Summary overview of trace elements and their daily dietary intake from various vegetables cultivated in the Bogra district of Bangladesh, along with the upper tolerable daily intake limit (UTIL).

Name of Vegetable	Toxic Metal	Contents of Trace Elements in Vegetables (µg g^−1^ Fresh wt.)								Mean Daily Intake of Toxic Metal (Mg Day^−1^ Person^−1^)	UTIL ^b^ (Mg Day^−1^ Person^−1^)
		Mean ^a^		Median		Maximum		Minimum			
		Shibganj	Kahaloo	Shibganj	Kahaloo	Shibganj	Kahaloo	Shibganj	Kahaloo		
Radish	Pb	1.94 ± 0.39 (12)	1.94 ± 1.22 (12)	0.67	2.11	6.22	3.38	<0.005	0.16	**0.325**	0.22 ^d^
	Ni	0.13 ± 0.02 (15)	0.51 ± 0.27 (12)	0.04	0.44	0.49	0.76	<0.010	0.38	0.047	1.00 ^c^
	Cd	0.004 ± 0.001 (9)	0.007 ± 0.003 (6)	0.001	0.007	0.012	0.016	<0.005	<0.005	0.001	0.06 ^d^
	Cr	1.79 ± 1.04 (18)	0.81 ± 0.61 (9)	1.12	0.70	5.58	1.87	0.10	<0.010	**0.235**	0.06 ^d^
Country bean	Pb	3.21 ± 1.07 (12)	5.24 ± 2.67 (12)	2.78	5.68	7.72	7.48	<0.005	2.14	**0.673**	0.22 ^d^
	Ni	0.45 ±0.35 (12)	0.71 ± 0.29 (12)	0.60	0.72	0.86	1.06	<0.010	0.32	0.093	1.00 ^c^
	Cd	0.001 ± 0.001 (6)	0.008 ± 0.004 (6)	0.00	0.004	0.003	0.023	<0.005	<0.005	0.001	0.06 ^d^
	Cr	3.14 ± 1.56 (15)	2.66 ± 1.23 (12)	2.86	2.44	6.67	5.38	<0.010	0.40	**0.493**	0.06 ^d^
Brinjal	Pb	2.08 ± 0.95 (12)	2.46 ± 0.86 (12)	2.19	2.66	4.23	4.45	<0.005	0.08	**0.374**	0.22 ^d^
	Ni	0.22 ± 0.21 (12)	0.41 ± 0.18 (12)	0.20	0.42	0.52	0.47	<0.010	0.32	0.049	1.00 ^c^
	Cd	0.024 ± 0.004 (15)	0.013 ± 0.001 (9)	0.009	0.014	0.106	0.025	<0.005	<0.005	0.003	0.06 ^d^
	Cr	2.28 ± 1.64 (18)	1.33 ± 0.67 (9)	2.32	0.97	3.97	3.39	0.25	<0.010	**0.318**	0.06 ^d^
Onion	Pb	5.06 ± 2.42 (15)	4.18 ± 2.98 (9)	2.13	4.91	21.80	6.88	<0.005	<0.005	**0.787**	0.22 ^d^
	Ni	0.23 ± 0.71 (15)	0.81 ± 0.30 (12)	0.21	0.58	0.53	2.04	<0.010	0.05	0.078	1.00 ^c^
	Cd	0.007 ± 0.003 (15)	0.016 ± 0.002 (6)	0.007	0.011	0.020	0.043	<0.005	<0.005	0.002	0.06 ^d^
	Cr	5.17 ± 2.46 (15)	1.75 ± 0.88 (12)	3.10	1.76	19.31	3.35	<0.010	0.14	**0.637**	0.06 ^d^
Chili	Pb	2.17 ± 1.93 (18)	7.86 ± 2.31 (12)	0.69	6.87	9.21	14.46	0.34	3.24	**0.744**	0.22 ^d^
	Ni	0.49 ± 0.46 (18)	0.66 ± 0.24 (12)	0.37	0.61	1.25	1.40	0.02	0.01	0.094	1.00 ^c^
	Cd	0.002 ± 0.01 (3)	0.016 ± 0.005 (6)	0.00	0.006	0.011	0.052	<0.005	<0.005	0.001	0.06 ^d^
	Cr	4.34± 1.19 (15)	2.07 ± 1.02 (9)	2.21	0.70	11.97	6.88	<0.010	<0.010	**0.574**	0.06 ^d^
Potato	Pb	5.19 ± 2.40 (18)	10.22 ± 1.28 (12)	3.86	8.34	9.36	18.58	2.48	5.61	**1.205**	0.22 ^d^
	Ni	0.62 ± 0.28 (18)	1.05 ± 0.10 (12)	0.64	0.98	1.19	1.77	0.02	0.47	0.133	1.00 ^c^
	Cd	0.003 ± 0.001 (3)	0.021 ± 0.003 (6)	0.00	0.012	0.017	0.062	<0.005	<0.005	0.002	0.06 ^d^
	Cr	0.83 ± 0.25 (9)	0.23 ± 0.02 (6)	0.28	0.20	3.62	0.49	<0.010	<0.010	**0.098**	0.06 ^d^

^a^ = Value in parenthesis indicates the number of samples that were above the limit of detection (LoD). ^b^ = Life stage group 19–50 years; ^c^ = FDA [63]; ^d^ = AMEC [64]. Bold values indicate a higher average daily intake of a specific toxic metal than the UTIL.

This study examines potatoes as a major daily staple in Bangladesh and focuses on potential health risks from toxic trace elements. People use potatoes practically every day to enhance meals and frequently combine them with meat, fish, and eggs for nutrition and taste. The study quantified Pb, Ni, Cd, and Cr concentrations in potatoes from farms in Bogra, finding 2.48–9.36, 0.02–1.19, <0.005–0.017, and <0.01–3.62 µg g^−1^, respectively, in the Shibganj subdistrict, and 5.61–18.58, 0.47–1.77, <0.005–0.062, and <0.01–0.49 µg g^−1^ in the Kahaloo subdistrict (Table 4). The results showed Pb and Ni in all samples from both subdistricts. However, some samples contained elevated Pb, Cd, and Ni levels, which may be causally linked to the abuse of agrochemicals during the growing season [4,7], as improper application can introduce these elements into crops. For regional context, Ud Din et al. [59] reported similar concentrations of Cr (2.12 µg g^−1^) and Ni (0.60 µg g^−1^), higher concentrations for Cd (0.66 µg g^−1^), and lower concentrations for Pb (2.23 µg g^−1^) in potatoes from Pakistan and Afghanistan. Likewise, Sarker et al. [65] found average Ni and Cd levels of 1.41 and 0.008 µg g^−1^, respectively, in potatoes from the Mymensingh district, with negligible Pb and Cr. In comparison, other studies in Bangladesh found higher trace element levels, which may be due to intentional sampling from contaminated areas [66] or various marketplaces [67], indicating the importance of sampling context in these findings. Finally, the present results showed that potatoes contribute the highest mean daily Pb intake—about six times the UTIL—raising health concerns (Table 4).

The study ranks the investigated vegetables by their toxic trace element levels and mean daily intake to identify key dietary sources of exposure. The mean daily intakes of Pb, Ni, Cd, and Cr from vegetables show these orders: potato > onion > chili > country bean > brinjal > radish for Pb; potato > chili > country bean > onion > brinjal > radish for Ni; brinjal > potato = onion > chili = country bean = radish for Cd; and onion > chili > country bean > brinjal > radish > potato for Cr (Table 4). Hence, it can be summarized that potatoes, onions, and chilies emerge as significant contributors of toxic trace elements. This information points out the need for increased awareness about the potential human health risks associated with these vegetables and emphasizes the importance of monitoring agricultural practices to mitigate trace metal contamination.

### 3.4. Bioconcentration of Trace Elements

The bioconcentration factor (BCF) was employed in the study to appraise the capacity of trace element transfer from farmlands to edible parts of vegetables. It is calculated by dividing the trace element concentration in the edible part of the vegetable (µg g^−1^, dry wt.) by its concentration in cultivated soils (µg g^−1^, dry wt.) [65,68]. Table 5 presents the calculated BCF values used to evaluate the ability of trace elements to accumulate in various vegetables from farm soil. The calculated BCF values demonstrated that for Pb accumulation, the studied vegetables in Shibganj ranked in the following order: onion > radish > potato > brinjal > country bean > chili, whereas in Kahaloo, the sequence was potato > chili > onion > country bean > brinjal > radish. As regards Ni, accumulation was higher in all vegetables in Kahaloo compared to Shibganj. The accumulation pattern for Kahaloo was radish > onion > potato > brinjal > country bean > chili, whereas for Shibganj, the order was potato > country bean > brinjal = chili > onion = radish. Table 5 shows that the calculated BCF values for Pb and Ni were < 1.0 in all six vegetables, indicating insignificant accumulation of these trace elements. On the other hand, the calculated average BCF value for Cd in brinjal collected from the Shibganj subdistrict was >1.0, but values ranged from 0.00 to 5.75; therefore, the mean above unity was driven by one high observation and should not be interpreted as consistent accumulation across all samples. The accumulation pattern of Cd among the vegetables studied showed the sequence brinjal > radish = onion > potato > chili > country bean in the Shibganj subdistrict, while in Kahaloo the order was brinjal > onion > radish > potato > chili > country bean. On the contrary, Cr had values >1.0 in both the subdistricts for all six vegetables except potato, indicating high accumulation of this trace element (Table 5). Furthermore, in most cases, accumulation was higher in all the vegetables in Shibganj than in Kahaloo. However, the accumulation pattern of Cr among the vegetables studied showed the sequence onion > brinjal > chili > radish > country bean > potato in the Shibganj subdistrict, whereas in Kahaloo the order was country bean > brinjal > onion > radish > chili > potato.

The calculated BCF patterns for Cd, Ni, and Pb are nearly identical to those reported earlier [65,69]. The data presented in Table 5 indicate that Cr and Cd have a greater ability to move from farmland to diverse vegetables grown in the Bogra area, as higher BCF values indicate greater accumulation of these trace elements in vegetables [68,70]. The study area’s soil physicochemical characteristics, especially its acidic pH, also favorably increase the bioavailability of trace elements. It is concerning that both subdistricts have comparatively higher BCF values for Cr and Cd. Compared to other metals, the highly toxic Cr(VI) form is readily available for uptake due to its high solubility and its ability to readily penetrate plant cells. Also, some plants actively move this ion into their cells using sulfate transporter pathways because it competes with sulfate ions that are needed [71]. However, Cd is a metal that is readily transported, readily absorbed by plant roots, and accumulates in the edible parts of vegetables [65]. According to Hart et al. [72], these divalent cations also compete with Ca since they have the same ion radius and charge. While the majority of trace elements can only enter plant tissues passively (for example, by diffusion), Ca can enter plant cells by active transport [73], and Cd can enter plant tissues through Ca channels [74]. However, soil characteristics, trace element availability and concentration, and plant species’ absorption and translocation capacities are among the factors affecting plant uptake of trace elements [68]. Conversely, Liu et al. [70] found that plant variety had a greater influence on trace element accumulation in crops than soil physicochemical properties or trace element levels. From the above results, it can be concluded that the accumulation of toxic trace elements in various vegetables varied significantly among locations. This suggests that environmental factors, along with soil characteristics, may contribute to such variations. However, understanding these differences can help in agricultural practices and food safety assessments.

### 3.5. Health Risk Assessment

#### 3.5.1. Non-Carcinogenic Risk

Summary statistics of non-carcinogenic human health risks due to dietary intake of toxic trace elements in various vegetables grown in two subdistricts of the Bogra district are presented in Table 6. Detailed data regarding the calculated chronic daily intake (CDI) and hazard quotient (HQ) are presented in Table S7. The measured HQ and hazard index (HI) values for adults due to dietary intake of various vegetables were below 1.0 for all toxic trace elements, except for potato, indicating that the amounts of trace metals in brinjal, radish, country bean, onion, and chili cultivated in the study regions were within the safe limit for non-carcinogenic human health risks. On the contrary, the calculated mean HQ value for Pb in potatoes collected from the Kahaloo subdistrict exceeded the threshold limit (<1.0), indicating that the amount of Pb in potato tubers posed non-carcinogenic human health concerns. However, Saha et al. [26] reported that the calculated HQ for all trace elements, including Pb, in potato tubers collected from the greater Mymensingh region were within the threshold limit. On the other hand, the mean measured HI values for adults in the Shibganj and Kahaloo subdistricts were 1.54 × 10^0^ and 2.09 × 10^0^, respectively. As regards the calculated HI, the risks from vegetables showed the sequence potato > brinjal > radish > onion > chili > country bean in Shibganj, while for Kahaloo the order was potato > brinjal > radish > country bean > chili > onion. However, the calculated ∑HI for an adult was 2.26 × 10^0^ and 2.59 × 10^0^ for the Shibganj and Kahaloo subdistricts, respectively (Table 6), indicating that HI values exceeded the threshold limit (<1.0). Therefore, dietary intake of these vegetables may be a serious health concern regarding food safety. Hence, additional, careful, and thorough analyses should be planned to pinpoint the sources of such elements in vegetables to enable the adoption of appropriate remediation strategies.

#### 3.5.2. Carcinogenic Risk

Cadmium (Cd) and Ni are categorized as group 1 carcinogens, while Pb is categorized as a group 2A potential human carcinogen by the International Agency for Research on Cancer (IARC). Such toxic elements increase the incidence of cancers by causing oxidative damage, disrupting gene transcription, and inducing cell death [75]. The measured incremental lifetime cancer risk (ILCR) scores for toxic elements have been used to estimate carcinogenic risks to human health from dietary exposure to various vegetables collected from two subdistricts of the Bogra district. The summary statistics for measured ILCR values for adults of Cd, Pb, and Ni are presented in Table 7, while detailed data are found in Table S8. According to USEPA [32], an ILCR score between 1.00 × 10^−6^ and 1.00 × 10^−4^ is considered bearable for cancer-related health concerns, while a risk of less than 1.00 × 10^−6^ is classified as negligible. The study results revealed that among the vegetables, radish, country bean, onion, and chili collected from all locations had ILCR values within a bearable range. But brinjal collected from one location in Shibganj had a higher ILCR value for Cd than the threshold limit. Similarly, two locations in each subdistrict also had higher cumulative ILCR (∑ILCR) values, indicating a higher cancer risk from dietary intake of brinjal produced in those locations (Table S8). However, this study recorded alarming results regarding potatoes. Samples collected from 1 location in Kahaloo for Pb; 5 and 4 locations in Shibganj and Kahaloo, respectively, for Ni; and 1 and 2 locations in Shibganj and Kahaloo, respectively, for Cd had ILCR values above the threshold limit, indicating cancer risk. As regards ∑ILCR values, 5 and 4 locations in Shibganj and Kahaloo, respectively, had greater ILCR values than the threshold limit due to dietary intake of potatoes grown in those locations, indicating cancer risks for adults (Table S8). However, based on ∑ILCR values, risks for vegetables showed the sequence potato > brinjal > radish > country bean > chili > onion in Shibganj, while in Kahaloo the order was potato > brinjal > radish > onion > country bean > chili.

Finally, the study highlights potential cancer risks due to dietary intake of toxic trace elements from certain vegetables—particularly potatoes and brinjal—grown in specific locations of two subdistricts in the Bogra district. Although risks for most vegetables are within a bearable ILCR range, higher ILCR values exceeding the threshold limits in various locations indicate that there must be continuous monitoring and mitigation strategies to ensure safe agricultural produce and protect public health.

### 3.6. Redundancy Analysis

**Figure 5:**
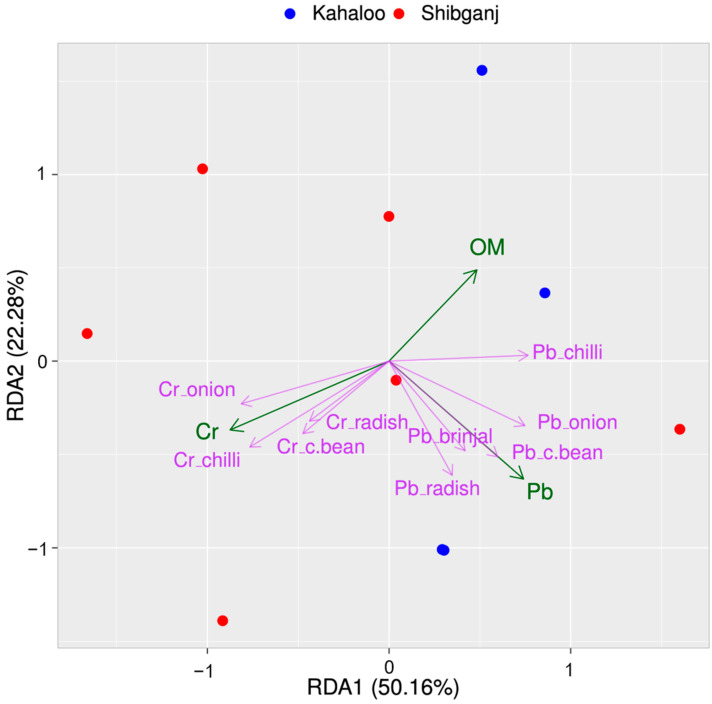
Redundancy analysis (RDA) showed the relationships between soil physicochemical properties (green lines) and trace element concentrations in vegetables (pink lines) collected from different farmers’ fields in the Shibganj and Kahaloo subdistricts of the Bogra district, Bangladesh.

Redundancy analysis (RDA) was performed to assess the relationships between soil physicochemical characteristics and levels of toxic trace elements in vegetables cultivated in two subdistricts of the Bogra district. Response variables were the levels of toxic trace elements in the vegetables. Soil features were considered explanatory factors. Prior to building the RDA model, the right-skewed trace element concentration data were log-transformed. A z-score transformation was used to normalize the soil variables. The model’s adjusted R^2^ was 0.706, indicating that approximately 70.6% of the variance in the response variables is explained by the model, suggesting a strong fit and robustness in capturing the relationships in the data. The first two RDA axes accounted for 72% of the variability, demonstrating that these components capture the majority of the meaningful patterns in the data. The variance inflation factor (VIF) of the RDA model for the soil parameters ranged from 1.55 to 3.71, indicating a permissible level of multicollinearity among the variables (Figure 5). The *p*-value (*p* = 0.001) for the overall model indicates a significant relationship between the response and explanatory variables. Among the explanatory variables, soil pH (*p* = 0.019), EC (*p* = 0.016), soil Cr (*p* = 0.001), and soil Pb concentrations (*p* = 0.011) had significant effects on the response variables, suggesting that these factors are particularly influential in shaping the observed outcomes. Finally, the data indicate that the measured soil parameters are strong predictors of the response variables, particularly soil pH, EC, Cr, and Pb concentrations. The low multicollinearity and strong model fit further strengthen confidence in these findings.

## 4. Limitations and Future Research Directions

Although the study made some insightful observations, it had several drawbacks. Firstly, it was limited to two subdistricts within the Bogra district, which may not be fully representative of other parts of the country. Additionally, the study considered four toxic trace elements (Pb, Cd, Ni, and Cr), as well as As and Hg, along with pesticide residues, which were not included due to a lack of analytical facilities at present. Furthermore, seasonal variation in trace element concentrations in soils and vegetables was not considered in this study, which limits understanding of temporal variations and fluctuations in trace metal content. Moreover, the analysis measured total Cr and did not distinguish Cr(III) from Cr(VI), and the risk calculations used total concentrations rather than the fraction released during human digestion. In addition, contamination indices were calculated against generalized shale values, which may not represent the natural geochemical background of Bogra. However, to better capture the spatial and temporal variability of contaminants in soils and vegetables, future studies should expand their scope to include other intensive vegetable-producing regions and account for multiple growing seasons and additional toxic substances. Furthermore, researchers should: (i) Establish local geochemical baselines using relatively undisturbed reference soils and depth profiles, (ii) Apply source-apportionment approaches by analyzing fertilizers, pesticides, irrigation water, road dust, and atmospheric deposition, (iii) Determine specific metal speciation—particularly Cr(VI)—rather than inferring toxicity from total chromium concentrations, and (iv) Utilize in vitro gastrointestinal bioaccessibility or bioavailability testing to refine dietary risk estimates.

## 5. Conclusions

Numerous trace element contaminations are progressively affecting Bangladesh’s agricultural land. Reports indicate elevated amounts of toxic trace elements in many agricultural products, including vegetables, fruits, and grains. This study measured Pb, Ni, Cd, and Cr contents in farm soils and six vegetables from two subdistricts of the Bogra district. The study identified Pb as the dominant soil contaminant and Pb and total Cr as the main dietary-exposure concerns in vegetables. Mean soil Pb concentrations were 158.3 and 164.1 µg g^−1^, approximately eight times the adopted average shale value of 20 µg g^−1^. All sites were classified as having moderate potential ecological risk, and PLI values exceeded 1.0 at 66.7% of Shibganj and 75.0% of Kahaloo sites. Estimated mean daily intakes of Pb and Cr ranged from 0.33 to 1.21 and 0.10 to 0.64 mg person^−1^ day^−1^, respectively, above the adopted tolerable intake limits. Dietary intake of potatoes showed both non-carcinogenic and carcinogenic risks, while brinjal showed only carcinogenic risks for adults. High levels of Pb and Cr in these frequently consumed vegetables threaten the local population; hence, continuous monitoring and mitigation are essential. The immediate, evidence-based priority is to identify and control the sources of Pb and Cr entering the soil–vegetable system. This should be achieved by: (i) Testing potential inputs: Evaluating fertilizers, pesticides, irrigation water, road or industrial dust, and background soils; and (ii) Implementing targeted management: Developing site-specific mitigation strategies or consumption guidance based on confirmed source apportionment, Cr speciation, and bioaccessibility data, rather than relying solely on total metal concentrations.

## Figures and Tables

**Table 1:** Statistical overview of the physicochemical properties of soils collected from vegetable-cultivating farmers’ fields in two subdistricts in the Bogra district, Bangladesh.

Parameter	Location (Mean ± SD)		Mean ± SD (*n* = 30)	Median (*n* = 30)	Maximum (*n* = 30)	Minimum (*n* = 30)
	Shibganj (*n* = 18)	Kahaloo (*n* = 12)				
pH	5.60 ± 0.48	4.89 ± 0.68	5.31 ± 0.57	5.43	6.43	4.42
EC (µS cm^−1^)	351.2 ± 75.2	195.5 ± 46.3	288.9 ± 61.0	280.7	462.0	160.2
OC (%)	0.81 ± 0.38	1.09 ± 0.38	0.92 ± 0.38	0.99	1.62	0.47
OM (%)	1.40 ± 0.66	1.89 ± 0.66	1.60 ± 0.66	1.71	2.79	0.82
Sand (%)	56.70 ± 4.46	56.54 ± 3.00	56.64 ± 3.74	57.04	63.04	51.04
Silt (%)	29.67 ± 3.14	28.00 ± 3.27	29.00 ± 3.22	28.00	33.00	24.00
Clay (%)	13.63 ± 1.75	15.46 ± 1.91	14.36 ± 1.85	13.46	16.96	11.96
Soil texture	Sandy loam-Loam	Sandy clay loam-Loam	-	-	-	-
Pb (µg g^−1^)	158.3 ± 8.83	164.1 ± 4.60	160.6 ± 0.63	160.4	171.0	148.1
Ni (µg g^−1^)	31.45 ± 5.25	35.69 ± 6.91	33.14 ± 0.44	31.25	42.65	27.09
Cd (µg g^−1^)	0.43 ± 0.08	0.53 ± 0.14	0.47 ± 0.03	0.46	0.67	0.32
Cr (µg g^−1^)	14.08 ± 2.16	9.37 ± 2.87	11.40 ± 0.74	11.55	23.01	4.32

**Table 2:** Comparative analysis of different toxic metals (in µg g^−1^) in vegetable-cultivating soils collected from farmers’ fields in the Bogra district, Bangladesh, with different reference values.

	Trace Metal Content (in µg g^−1^)			
	Pb	Ni	Cd	Cr
Present study average	160.6 ± 0.63	33.1 ± 0.44	0.47 ± 0.03	11.4 ± 0.74
Average shale value ^a^	20	68	0.30	90
Crustal average ^b^	12.5	75	0.20	100
Earth’s crust (upper continental crust) ^c^	16	58	0.13	83
USEPA toxicity reference value (TRV) ^d^	100	100	10.0	-
Canadian soil quality guideline ^e^	70	50	1.40	64
Soil quality guidelines- Netherlands ^f^	85	35	0.80	100

^a^ = Turekian and Wedepohl [25]; ^b^ = Taylor [48]; ^c^ = Yaroshevsky [49]; ^d^ = USEPA [50]; ^e^ = CCME [51]; ^f^ = Swartjes et al. [52].

**Table 3:** Summary statistics of contamination factor (CF), pollution load index (PLI), single potential ecological risk factor (SPERF) for toxic metals and potential ecological risk index (PERI) for different vegetables cultivating soils of the Bogra district in Bangladesh.

Name of Subdistrict	Sample ID	CF Value for Toxic Metals				PLI	SPERF Value for Toxic Metals				PERI
		Cd	Pb	Ni	Cr		Cd	Pb	Ni	Cr	
Shibganj	Mean	2.15	12.66	0.42	0.14	1.02	64.40	63.31	2.10	0.26	130.06
	Median	2.15	12.65	0.39	0.13	1.10	64.35	63.23	1.95	0.26	127.93
	Maximum	2.78	13.68	0.53	0.23	1.32	83.40	68.40	2.63	0.46	145.15
	Minimum	1.60	11.85	0.36	0.04	0.56	48.00	59.25	1.81	0.02	119.04
Kahaloo	Mean	2.66	13.13	0.48	0.09	1.10	79.88	65.63	2.38	0.19	148.07
	Median	2.76	13.09	0.48	0.09	1.08	82.65	65.45	2.41	0.18	151.04
	Maximum	3.36	13.57	0.57	0.13	1.30	100.80	67.85	2.85	0.25	170.22
	Minimum	1.78	12.75	0.37	0.07	0.96	53.40	63.75	1.86	0.14	119.97

**Table 5:** Summary statistics of calculated bioconcentration factor (BCF) of trace elements for various vegetables cultivated in the Bogra district of Bangladesh.

Name of Vegetable	Toxic Metal	BCF Values of Trace Elements in Vegetables							
		Mean		Median		Maximum		Minimum	
		Shibganj	Kahaloo	Shibganj	Kahaloo	Shibganj	Kahaloo	Shibganj	Kahaloo
Radish	Pb	0.29	0.29	0.11	0.32	0.91	0.51	0.00	0.02
	Ni	0.10	0.35	0.03	0.33	0.33	0.44	0.00	0.30
	Cd	0.24	0.29	0.07	0.28	0.74	0.59	0.00	0.00
	Cr	**2.42**	**1.87**	1.98	1.87	6.11	3.74	0.48	0.00
Country bean	Pb	0.22	0.35	0.19	0.38	0.50	0.50	0.00	0.15
	Ni	0.15	0.21	0.21	0.22	0.26	0.29	0.00	0.11
	Cd	0.02	0.13	0.00	0.08	0.06	0.37	0.00	0.00
	Cr	**2.21**	**2.91**	2.48	3.11	3.53	4.79	0.00	0.64
Brinjal	Pb	0.25	0.30	0.27	0.32	0.49	0.54	0.00	0.01
	Ni	0.13	0.23	0.10	0.22	0.34	0.32	0.00	0.19
	Cd	**1.23**	0.46	0.39	0.50	5.75	0.83	0.00	0.00
	Cr	**2.88**	**2.73**	2.82	2.74	4.85	5.43	1.17	0.00
Onion	Pb	0.43	0.36	0.19	0.42	1.82	0.59	0.00	0.00
	Ni	0.10	0.29	0.11	0.22	0.19	0.68	0.00	0.03
	Cd	0.24	0.35	0.20	0.25	0.68	0.91	0.00	0.00
	Cr	**3.85**	**2.23**	3.81	2.27	8.95	4.09	0.00	0.28
Chilli	Pb	0.12	0.44	0.04	0.38	0.49	0.81	0.02	0.18
	Ni	0.13	0.15	0.10	0.14	0.29	0.32	0.01	0.00
	Cd	0.03	0.22	0.00	0.09	0.18	0.70	0.00	0.00
	Cr	**2.45**	**1.71**	2.54	0.62	4.77	5.62	0.00	0.00
Potato	Pb	0.27	0.52	0.21	0.41	0.46	0.96	0.14	0.29
	Ni	0.16	0.25	0.17	0.20	0.25	0.46	0.01	0.14
	Cd	0.04	0.27	0.00	0.15	0.26	0.77	0.00	0.00
	Cr	0.32	0.16	0.14	0.15	1.32	0.33	0.00	0.00

Mean bold values indicate significant accumulation of a particular toxic element.

**Table 6:** Calculated summary overview of non-carcinogenic human health risks due to dietary intake of toxic metals from various vegetables cultivated in the Bogra district of Bangladesh.

Name of Vegetable	Descriptive Statistics	Hazard Quotient (HQ) Values								Hazard Index (HI) Values	
		Pb		Ni		Cd		Cr			
		Shibganj	Kahaloo	Shibganj	Kahaloo	Shibganj	Kahaloo	Shibganj	Kahaloo	Shibganj	Kahaloo
Radish	Mean	2.27 × 10^−2^	2.26 × 10^−2^	2.71 × 10^−4^	1.06 × 10^−3^	1.75 × 10^−4^	3.09 × 10^−4^	7.53 × 10^−2^	3.42 × 10^−2^	9.84 × 10^−2^	5.82 × 10^−2^
	Median	7.81 × 10^−3^	2.46 × 10^−2^	7.92 × 10^−5^	9.32 × 10^−4^	5.08 × 10^−5^	2.84 × 10^−4^	4.71 × 10^−2^	2.92 × 10^−2^	8.40 × 10^−2^	5.15 × 10^−2^
	Maximum	7.26 × 10^−2^	3.94 × 10^−2^	1.03 × 10^−3^	1.59 × 10^−3^	4.91 × 10^−4^	6.68 × 10^−4^	2.34 × 10^−1^	7.84 × 10^−2^	2.83 × 10^−1^	1.11 × 10^−1^
	Minimum	0.00 × 10^0^	1.85 × 10^−3^	0.00 × 10^0^	7.93 × 10^−4^	0.00 × 10^0^	0.00 × 10^0^	4.02 × 10^−3^	0.00 × 10^0^	4.12 × 10^−3^	1.91 × 10^−2^
Brinjal	Mean	8.46 × 10^−2^	1.00 × 10^−1^	1.61 × 10^−3^	2.99 × 10^−3^	3.49 × 10^−3^	1.95 × 10^−3^	3.34 × 10^−1^	1.95 × 10^−1^	4.24 × 10^−1^	3.01 × 10^−1^
	Median	8.90 × 10^−2^	1.08 × 10^−1^	1.47 × 10^−3^	3.08 × 10^−3^	1.37 × 10^−3^	2.05 × 10^−3^	3.39 × 10^−1^	1.43 × 10^−1^	4.02 × 10^−1^	2.68 × 10^−1^
	Maximum	1.72 × 10^−1^	1.81 × 10^−1^	3.79 × 10^−3^	3.46 × 10^−3^	1.55 × 10^−2^	3.71 × 10^−3^	5.82 × 10^−1^	4.96 × 10^−1^	6.69 × 10^−1^	6.59 × 10^−1^
	Minimum	0.00 × 10^0^	3.45 × 10^−3^	0.00 × 10^0^	2.33 × 10^−3^	0.00 × 10^0^	0.00 × 10^0^	3.70 × 10^−2^	0.00 × 10^0^	1.94 × 10^−1^	7.85 × 10^−3^
Country bean	Mean	1.23 × 10^−2^	2.00 × 10^−2^	3.12 × 10^−4^	4.85 × 10^−4^	1.17 × 10^−5^	1.07 × 10^−4^	4.31 × 10^−2^	3.66 × 10^−2^	5.57 × 10^−2^	5.72 × 10^−2^
	Median	1.06 × 10^−2^	2.17 × 10^−2^	4.11 × 10^−4^	4.95 × 10^−4^	0.00 × 10^0^	5.98 × 10^−5^	3.93 × 10^−2^	3.35 × 10^−2^	5.27 × 10^−2^	5.26 × 10^−2^
	Maximum	2.95 × 10^−2^	2.86 × 10^−2^	5.94 × 10^−4^	7.30 × 10^−4^	4.13 × 10^−5^	3.09 × 10^−4^	9.16 × 10^−2^	7.39 × 10^−2^	9.53 × 10^−2^	1.01 × 10^−1^
	Minimum	0.00 × 10^0^	8.16 × 10^−3^	0.00 × 10^0^	2.20 × 10^−4^	0.00 × 10^0^	0.00 × 10^0^	0.00 × 10^0^	5.48 × 10^−3^	1.45 × 10^−2^	2.27 × 10^−2^
Onion	Mean	1.76 × 10^−2^	1.45 × 10^−2^	1.45 × 10^−4^	5.08 × 10^−4^	9.04 × 10^−5^	2.00 × 10^−4^	6.47 × 10^−2^	2.19 × 10^−2^	8.25 × 10^−2^	3.71 × 10^−2^
	Median	7.40 × 10^−3^	1.70 × 10^−2^	1.30 × 10^−4^	3.61 × 10^−4^	8.71 × 10^−5^	1.33 × 10^−4^	3.88 × 10^−2^	2.21 × 10^−2^	5.24 × 10^−2^	3.91 × 10^−2^
	Maximum	7.57 × 10^−2^	2.39 × 10^−2^	3.31 × 10^−4^	1.28 × 10^−3^	2.50 × 10^−4^	5.33 × 10^−4^	2.41 × 10^−1^	4.18 × 10^−2^	2.44 × 10^−1^	6.55 × 10^−2^
	Minimum	0.00 × 10^0^	0.00 × 10^0^	0.00 × 10^0^	3.34 × 10^−5^	0.00 × 10^0^	0.00 × 10^0^	0.00 × 10^0^	1.70 × 10^−3^	2.92 × 10^−2^	4.86 × 10^−3^
Chilli	Mean	7.03 × 10^−3^	2.55 × 10^−2^	2.87 × 10^−4^	3.83 × 10^−4^	2.12 × 10^−5^	1.85 × 10^−4^	5.06 × 10^−2^	2.41 × 10^−2^	5.80 × 10^−2^	5.02 × 10^−2^
	Median	2.24 × 10^−3^	2.23 × 10^−2^	2.14 × 10^−4^	3.57 × 10^−4^	0.00 × 10^0^	6.85 × 10^−5^	2.57 × 10^−2^	8.13 × 10^−3^	3.04 × 10^−2^	4.09 × 10^−2^
	Maximum	2.98 × 10^−2^	4.69 × 10^−2^	7.28 × 10^−4^	8.17 × 10^−4^	1.27 × 10^−4^	6.05 × 10^−4^	1.40 × 10^−1^	8.03 × 10^−2^	1.45 × 10^−1^	9.19 × 10^−2^
	Minimum	1.11 × 10^−3^	1.05 × 10^−2^	9.43 × 10^−6^	2.91 × 10^−6^	0.00 × 10^0^	0.00 × 10^0^	0.00 × 10^0^	0.00 × 10^0^	1.20 × 10^−2^	2.71 × 10^−2^
Potato	Mean	9.61 × 10^−1^	**1.89 × 10^0^**	2.07 × 10^−2^	3.50 × 10^−2^	1.93 × 10^−3^	1.41 × 10^−2^	5.52 × 10^−1^	1.50 × 10^−1^	**1.54 × 10^0^**	**2.09 × 10^0^**
	Median	7.16 × 10^−1^	**1.54 × 10^0^**	2.12 × 10^−2^	3.26 × 10^−2^	0.00 × 10^0^	7.57 × 10^−3^	1.85 × 10^−1^	1.36 × 10^−1^	**1.01 × 10^0^**	**1.91 × 10^0^**
	Maximum	**1.73 × 10^0^**	**3.44 × 10^0^**	3.95 × 10^−2^	5.89 × 10^−2^	1.16 × 10^−2^	4.13 × 10^−2^	**2.42 × 10^0^**	3.28 × 10^−1^	**4.05 × 10^0^**	**3.47 × 10^0^**
	Minimum	4.60 × 10^−1^	**1.04 × 10^0^**	7.45 × 10^−4^	1.57 × 10^−2^	0.00 × 10^0^	0.00 × 10^0^	0.00 × 10^0^	0.00 × 10^0^	5.18 × 10^−1^	**1.07 × 10^0^**
									∑HI	**2.26 × 10^0^**	**2.59 × 10^0^**

Bold values indicate non-carcinogenic human health risks due to dietary intake of toxic metals.

**Table 7:** Calculated summary overview of carcinogenic human health risks due to dietary intake of toxic metals from various vegetables cultivated in the Bogra district of Bangladesh.

Name of Vegetable	Descriptive Statistics	Incremental Lifetime Cancer Risk (ILCR)						Total ILCR (∑ILCR)	
		Pb		Ni		Cd			
		Shibganj	Kahaloo	Shibganj	Kahaloo	Shibganj	Kahaloo	Shibganj	Kahaloo
Radish	Mean	6.93 × 10^−7^	6.91 × 10^−7^	4.94 × 10^−6^	1.93 × 10^−5^	2.63 × 10^−6^	4.64 × 10^−6^	8.26 × 10^−6^	2.46 × 10^−5^
	Median	2.39 × 10^−7^	7.52 × 10^−7^	1.44 × 10^−6^	1.70 × 10^−5^	7.62 × 10^−7^	4.27 × 10^−6^	7.80 × 10^−6^	2.15 × 10^−5^
	Maximum	2.22 × 10^−6^	1.21 × 10^−6^	1.87 × 10^−5^	2.89 × 10^−5^	7.36 × 10^−6^	1.00 × 10^−5^	2.02 × 10^−5^	4.01 × 10^−5^
	Minimum	0.00 × 10^0^	5.65 × 10^−8^	0.00 × 10^0^	1.44 × 10^−5^	0.00 × 10^0^	0.00 × 10^0^	1.78 × 10^−6^	1.54 × 10^−5^
Brinjal	Mean	2.59 × 10^−6^	3.07 × 10^−6^	2.92 × 10^−5^	5.44 × 10^−5^	5.24 × 10^−5^	2.92 × 10^−5^	8.42 × 10^−5^	8.67 × 10^−5^
	Median	2.72 × 10^−6^	3.31 × 10^−6^	2.67 × 10^−5^	5.60 × 10^−5^	2.05 × 10^−5^	3.07 × 10^−5^	7.48 × 10^−5^	9.15 × 10^−5^
	Maximum	5.26 × 10^−6^	5.54 × 10^−6^	6.89 × 10^−5^	6.30 × 10^−5^	**2.32 × 10^−4^**	5.56 × 10^−5^	**2.36 × 10^−4^**	**1.16 × 10^−4^**
	Minimum	0.00 × 10^0^	1.05 × 10^−7^	0.00 × 10^0^	4.25 × 10^−5^	0.00 × 10^0^	0.00 × 10^0^	4.82 × 10^−6^	4.74 × 10^−5^
Country bean	Mean	3.75 × 10^−7^	6.13 × 10^−7^	5.67 × 10^−6^	8.83 × 10^−6^	1.75 × 10^−7^	1.61 × 10^−6^	6.22 × 10^−6^	1.11 × 10^−5^
	Median	3.25 × 10^−7^	6.64 × 10^−7^	7.48 × 10^−6^	9.02 × 10^−6^	0.00 × 10^0^	8.97 × 10^−7^	8.28 × 10^−6^	1.10 × 10^−5^
	Maximum	9.02 × 10^−7^	8.74 × 10^−7^	1.08 × 10^−5^	1.33 × 10^−5^	6.19 × 10^−7^	4.64 × 10^−6^	1.08 × 10^−5^	1.73 × 10^−5^
	Minimum	0.00 × 10^0^	2.50 × 10^−7^	0.00 × 10^0^	4.00 × 10^−6^	0.00 × 10^0^	0.00 × 10^0^	7.52 × 10^−8^	4.83 × 10^−6^
Onion	Mean	5.37 × 10^−7^	4.44 × 10^−7^	2.65 × 10^−6^	9.24 × 10^−6^	1.36 × 10^−6^	3.00 × 10^−6^	4.54 × 10^−6^	1.27 × 10^−5^
	Median	2.26 × 10^−7^	5.22 × 10^−7^	2.36 × 10^−6^	6.57 × 10^−6^	1.31 × 10^−6^	2.00 × 10^−6^	3.56 × 10^−6^	7.51 × 10^−6^
	Maximum	2.32 × 10^−6^	7.31 × 10^−7^	6.02 × 10^−6^	2.32 × 10^−5^	3.75 × 10^−6^	7.99 × 10^−6^	8.67 × 10^−6^	3.19 × 10^−5^
	Minimum	0.00 × 10^0^	0.00 × 10^0^	0.00 × 10^0^	6.09 × 10^−7^	0.00 × 10^0^	0.00 × 10^0^	1.36 × 10^−6^	3.76 × 10^−6^
Chilli	Mean	2.15 × 10^−7^	7.80 × 10^−7^	5.23 × 10^−6^	6.98 × 10^−6^	3.18 × 10^−7^	2.78 × 10^−6^	5.76 × 10^−6^	1.05 × 10^−5^
	Median	6.85 × 10^−8^	6.81 × 10^−7^	3.89 × 10^−6^	6.50 × 10^−6^	0.00 × 10^0^	1.03 × 10^−6^	3.94 × 10^−6^	9.79 × 10^−6^
	Maximum	9.13 × 10^−7^	1.43 × 10^−6^	1.33 × 10^−5^	1.49 × 10^−5^	1.91 × 10^−6^	9.07 × 10^−6^	1.42 × 10^−5^	1.90 × 10^−5^
	Minimum	3.41 × 10^−8^	3.22 × 10^−7^	1.72 × 10^−7^	5.30 × 10^−8^	0.00 × 10^0^	0.00 × 10^0^	2.52 × 10^−7^	3.54 × 10^−6^
Potato	Mean	2.94 × 10^−5^	5.79 × 10^−5^	**3.78 × 10^−4^**	**6.36 × 10^−4^**	2.90 × 10^−5^	**2.12 × 10^−4^**	**4.36 × 10^−4^**	**9.06 × 10^−4^**
	Median	2.19 × 10^−5^	4.72 × 10^−5^	**3.86 × 10^−4^**	**5.93 × 10^−4^**	0.00 × 10^0^	**1.14 × 10^−4^**	**4.07 × 10^−4^**	**8.75 × 10^−4^**
	Maximum	5.30 × 10^−5^	**1.05 × 10^−4^**	**7.19 × 10^−4^**	**1.07 × 10^−3^**	**1.74 × 10^−4^**	**6.20 × 10^−4^**	**7.72 × 10^−4^**	**1.37 × 10^−3^**
	Minimum	1.41 × 10^−5^	3.18 × 10^−5^	1.36 × 10^−5^	**2.86 × 10^−4^**	0.00 × 10^0^	0.00 × 10^0^	6.37 × 10^−5^	**4.99 × 10^−4^**

Bold values indicate carcinogenic human health risks due to dietary intake of toxic metals.

## Data Availability

The original contributions presented in this study are included in the article and Supplementary Tables. Further inquiries can be directed to the corresponding author.

## Supplementary Materials

The following supporting information can be downloaded at https://www.mdpi.com/article/10.3390/jox16040127/s1: Please see “Supplementary Tables” attached with the manuscript.

## Abbreviations

The following abbreviations are used in this manuscript:

AAS	Atomic absorption spectrophotometer
BCF	Bioconcentration factor
CDI	Chronic daily intake
CF	Contamination factor
CRM	Certified reference material
CSF	Cancer slope factor
HI	Hazard index
HQ	Hazard quotient
IARC	International Agency for Research on Cancer
ILCR	Incremental lifetime cancer risk
PERI	Potential ecological risk index
PLI	Pollution load index
SPERF	Single potential ecological risk factors
RDA	Redundancy analysis
RfD	Oral reference dose
USEPA	United States Environmental Protection Agency
UTIL	Upper tolerable intake limit
