# Supplementary material for: Potential Toxic Elements in Farm Soils and Vegetables of Northern Bangladesh: Impact on Soil Health and Human Safety
Source: J Xenobiot. 2026 Jul 10;16(4):127. doi: 10.3390/jox16040127 (PMC13398253; doi:10.3390/jox16040127)
Supplement: Supplementary file 1 [file jox-16-00127-s001.zip › jox-4405950-supplementary.pdf]

Suppl. Table S1: Details of operating parameters for determination of toxic heavy metals using atomic absorption spectrophotometer (AAS); (Model: Shimadzu AA-7000)

Trace metals	Analytical wavelength (nm)	Minimum concentration limit of detection ($\mu g\ g^{-1}$)	The concentration of the standard series solution used in calibration ($\mu g\ mL^{-1}$)	Obtained equation with R^2 value
Pb	217.0	0.005	0.0, 1.0, 2.0, 3.0, 4.0 & 5.0	$Y = 0.0175x - 0.0113$; $R^2 = 0.998$
Cd	228.8	0.005	0.0, 0.1, 0.2, 0.4 & 0.8	$Y = 0.5669x + 0.0043$; $R^2 = 0.999$
Ni	232.0	0.01	0.0, 1.0, 2.0 & 4.0	$Y = 0.0205x + 0.0007$; $R^2 = 0.997$
Cr	357.9	0.01	0.0, 0.5, 1.0, 2.0 & 4.0	$Y = 0.0898x + 0.0162$; $R^2 = 0.998$

Suppl. Table S2: Analytical results obtained for trace elements present in Certified Reference Materials (CRM) along with their percent recovery

Trace metals	JLk-1 (Lake sediment)		
	Certified value (μg g^{-1})	Observed value (μg g^{-1})	Recovery (%)
Pb	43.70	44.36±2.83	101.5
Ni	35.00	33.44±1.46	95.5
Cd	0.572	0.588±0.10	102.8
Cr	69.00	63.42±2.26	91.9

Suppl. Table S3: Average toxic metal concentrations along with other physicochemical properties in vegetable producing farmers field soils of Bogra district in Bangladesh

Sample ID	pH	EC ($\mu S/cm$)	OC (%)	OM (%)	Sand (%)	Silt (%)	Clay (%)	Textural Class	Cr (µg g^{-1})	Cd (µg g^{-1})	Ni (µg g^{-1})	Pb (µg g^{-1})
1	6.43±0.43	306.0±68.4	0.47±0.26	0.82±0.44	51.04±4.43	32.0±2.49	16.96±1.29	Loam	23.01±0.87	0.556±0.24	30.49±0.53	148.1±0.56
2	5.58±0.52	411.0±82.0	1.09±0.50	1.90±0.86	59.04±5.48	28.0±3.79	12.96±1.75	Sandy Loam	13.58±1.03	0.423±0.01	36.47±0.35	149.4±0.99
3	5.29±0.48	367.0±75.2	0.47±0.38	0.82±0.65	53.04±3.44	33.0±3.14	13.96±1.94	Loam	22.82±0.76	0.368±0.01	27.09±0.69	164.9±0.50
4	5.02±0.44	462.0±79.0	0.47±0.46	0.82±0.79	63.04±4.49	25.0±2.62	11.96±1.56	Sandy Loam	4.32±0.60	0.436±0.01	28.02±0.31	156.7±1.52
5	5.57±0.53	290.6±71.4	1.30±0.30	2.26±0.52	55.04±5.11	32.0±3.66	12.96±1.75	Sandy Loam	11.97±1.16	0.475±0.02	27.22±0.24	159.4±1.00
6	5.68±0.48	270.8±75.2	1.04±0.38	1.79±0.65	59.04±3.81	28.0±3.14	12.96±2.21	Sandy Loam	0.88±0.02	0.320±0.01	39.40±0.46	171.0±0.58
7	4.67±0.76	160.2±46.3	1.08±0.45	1.87±0.77	55.04±2.35	28.0±4.11	16.96±2.13	Sandy Loam	6.93±0.76	0.612±0.02	27.89±0.53	161.4±0.02
8	4.58±0.67	172.0±56.1	1.62±0.37	2.79±0.64	59.04±3.20	28.0±3.27	12.96±1.91	Sandy Loam	6.93±0.01	0.355±0.01	40.19±0.64	159.4±0.01
9	5.90±0.60	186.8±36.5	0.94±0.31	1.63±0.53	59.04±2.81	24.0±2.43	16.96±1.69	Sandy Loam	12.48±1.58	0.491±0.01	32.01±0.23	169.7±0.57
10	4.42±0.69	263.0±46.3	0.72±0.39	1.25±0.67	53.04±3.65	32.0±3.27	14.96±1.91	Loam	11.13±0.59	0.671±0.01	42.65±0.34	165.8±0.50
Mean	5.31±0.57	288.94±61.0	0.92±0.38	1.60±0.66	56.64±3.74	29.00±3.22	14.36±1.85	-	11.40±0.74	0.47±0.03	33.14±0.44	160.58±0.63
Median	5.43	280.70	0.99	1.71	57.04	28.00	13.46	-	11.55	0.46	31.25	160.38
Max.	6.43	462.00	1.62	2.79	63.04	33.00	16.96	-	23.01	0.67	42.65	171.00
Min.	4.42	160.20	0.47	0.82	51.04	24.00	11.96	-	0.88	0.32	27.09	148.12

Sample IDs 1-6 and 7-10 represent locations of Shibganj and Kahaloo, respectively.

Suppl. Table S4: Calculated mean values of contamination factor (CF), pollution load index (PLI), and potential ecological risk index (PERI) for different vegetables cultivating soils of Bogra district in Bangladesh

Sample ID	CF value for toxic trace metals				PLI	SPERF value for toxic metals				PERI
	**Cr**	**Cd**	**Pb**	**Ni**		**Cr**	**Cd**	**Pb**	**Ni**	
1	0.230	2.78	11.85	0.407	1.32	0.46	83.40	59.25	2.04	145.15
2	0.136	2.11	11.96	0.486	1.14	0.27	63.30	59.80	2.43	125.80
3	0.228	1.84	13.19	0.361	1.19	0.46	55.20	65.95	1.81	123.41
4	0.043	2.18	12.54	0.374	0.81	0.09	65.40	62.70	1.87	130.06
5	0.120	2.37	12.75	0.363	1.07	0.24	71.10	63.75	1.82	136.91
6	0.009	1.60	13.68	0.525	0.56	0.02	48.00	68.40	2.63	119.04
7	0.069	3.06	12.91	0.372	1.00	0.14	91.80	64.55	1.86	158.35
8	0.069	1.78	12.75	0.536	0.96	0.14	53.40	63.75	2.68	119.97
9	0.125	2.45	13.57	0.427	1.15	0.25	73.50	67.85	2.14	143.74
10	0.111	3.36	13.27	0.569	1.30	0.22	100.80	66.35	2.85	170.22
Mean	0.114	2.353	12.846	0.442	1.051	0.23	70.59	64.24	2.21	137.26
Median	0.116	2.278	12.831	0.417	1.104	0.23	68.25	64.15	2.09	133.48
Max.	0.230	3.357	13.680	0.569	1.325	0.46	100.80	68.40	2.85	170.22
Min.	0.009	1.602	11.849	0.361	0.564	0.02	48.00	59.25	1.81	119.04

Sample IDs 1-6 and 7-10 represent locations of Shibganj and Kahaloo, respectively.

Suppl. Table S5: Average toxic metals concentration in various vegetables cultivated in Bogra district of Bangladesh

Sample ID	Radish				Brinjal				Country bean			
	Cr (µg g^{-1})	Cd (µg g^{-1})	Ni (µg g^{-1})	Pb (µg g^{-1})	Cr (µg g^{-1})	Cd (µg g^{-1})	Ni (µg g^{-1})	Pb (µg g^{-1})	Cr (µg g^{-1})	Cd (µg g^{-1})	Ni (µg g^{-1})	Pb (µg g^{-1})
1	2.612±0.07	0.0117±0.001	0.000±0.00	0.165±0.102	3.973±0.06	0.0145±0.001	0.517±0.014	0.000±0.00	6.665±0.11	0.0030±0.001	0.661±0.019	0.000±0.00
2	1.845±0.00	0.0024±0.004	0.488±0.001	0.000±0.00	3.294±0.06	0.0136±0.003	0.398±0.004	0.000±0.00	1.919±0.10	0.0000±0.00	0.864±0.017	0.000±0.00
3	5.580±0.03	0.0110±0.001	0.029±0.001	4.090±0.014	3.878±0.01	0.1058±0.004	0.006±0.001	2.090±0.002	5.564±0.06	0.0021±0.001	0.000±0.00	4.912±0.094
4	0.096±0.01	0.0000±0.00	0.047±0.002	0.000±0.00	0.252±0.03	0.0000±0.00	0.000±0.00	3.868±0.001	0.875±0.22	0.0000±0.00	0.000±0.00	0.643±0.00
5	0.231±0.04	0.0000±0.00	0.203±0.010	1.175±0.024	1.337±0.10	0.0051±0.001	0.000±0.00	2.285±0.019	3.803±0.05	0.0000±0.00	0.583±0.008	5.980±0.072
6	0.398±0.07	0.0000±0.00	0.008±0.006	6.220±0.129	0.957±0.07	0.0040±0.001	0.395±0.015	4.227±0.032	0.000±0.00	0.0000±0.00	0.612±0.029	7.718±0.032
7	0.000±0.00	0.0135±0.001	0.404±0.005	1.520±0.023	1.815±0.09	0.0253±0.002	0.443±0.019	1.384±0.065	2.225±0.11	0.0087±0.002	0.499±0.011	2.136±0.152
8	0.447±0.03	0.0000±0.00	0.484±0.001	0.158±0.001	0.000±0.00	0.0065±0.001	0.473±0.004	0.085±0.003	0.398±0.02	0.0000±0.00	1.061±0.025	4.329±0.112
9	1.867±0.04	0.0000±0.00	0.378±0.010	2.692±0.019	3.387±0.03	0.0000±0.00	0.319±0.007	3.939±0.019	5.377±0.07	0.0000±0.00	0.320±0.008	7.032±0.084
10	0.946±0.05	0.0159±0.002	0.756±0.017	3.376±0.032	0.131±0.05	0.0215±0.002	0.397±0.022	4.452±0.084	2.651±0.01	0.0225±0.003	0.942±0.008	7.479±0.084
Mean	1.40±0.03	0.0054±0.001	0.28±0.005	1.94±0.034	1.90±0.05	0.0196±0.002	0.29±0.009	2.23±0.023	2.95±0.08	0.0036±0.001	0.55±0.013	4.02±0.063
Median	0.70	0.0012	0.29	1.35	1.58	0.0100	0.40	2.19	2.44	0.0000	0.60	4.62
Maximum	5.58	0.0159	0.76	6.22	3.97	0.1058	0.52	4.45	6.67	0.0225	1.06	7.72
Minimum	0.00	0.000	0.00	0.00	0.00	0.0000	0.00	0.00	0.00	0.0000	0.00	0.00

Sample ID	Onion				Chilli				Potato			
	Cr (µg g^{-1})	Cd (µg g^{-1})	Ni (µg g^{-1})	Pb (µg g^{-1})	Cr (µg g^{-1})	Cd (µg g^{-1})	Ni (µg g^{-1})	Pb (µg g^{-1})	Cr (µg g^{-1})	Cd (µg g^{-1})	Ni (µg g^{-1})	Pb (µg g^{-1})
1	19.311±0.06	0.0074±0.00	0.024±0.016	0.763±0.023	8.787±0.06	0.0109±0.001	0.920±0.009	0.344±0.047	0.788±0.10	0.0174±0.007	0.792±0.024	2.826±0.060
2	4.296±0.14	0.0200±0.001	0.429±0.001	0.385±0.033	2.415±0.12	0.0000±0.00	0.533±0.012	0.568±0.051	0.555±0.11	0.0000±0.00	0.733±0.025	2.481±0.046
3	3.246±0.06	0.0073±0.001	0.000±0.00	0.000±0.00	11.97±0.10	0.0000±0.00	0.039±0.005	1.516±0.001	3.624±0.07	0.0000±0.00	0.022±0.00	8.842±0.106
4	1.233±0.05	0.0067±0.001	0.233±0.004	3.895±0.019	1.998±0.00	0.0000±0.00	0.016±0.002	0.814±0.001	0.000±0.00	0.0000±0.00	0.461±0.001	2.714±0.078
5	2.962±0.02	0.0020±0.00	0.182±0.011	3.497±0.068	0.860±0.03	0.0000±0.00	0.199±0.024	0.566±0.088	0.000±0.00	0.0000±0.00	0.539±0.013	4.903±0.263
6	0.000±0.00	0.0000±0.00	0.529±0.010	21.79±0.149	0.000±0.00	0.0000±0.00	1.249±0.032	9.207±0.105	0.000±0.00	0.0000±0.00	1.186±0.048	9.360±0.112
7	0.136±0.03	0.0213±0.001	0.054±0.006	4.222±0.023	0.368±0.39	0.0117±0.002	0.005±0.002	14.46±0.173	0.000±0.00	0.0227±0.001	0.471±0.008	18.58±0.146
8	0.345±0.00	0.0000±0.00	0.876±0.001	0.000±0.00	0.000±0.00	0.0000±0.00	1.401±0.003	9.139±0.014	0.000±0.00	0.0000±0.00	0.770±0.042	5.613±0.001
9	3.348±0.04	0.0000±0.00	0.278±0.013	5.597±0.032	1.026±0.01	0.0000±0.00	0.315±0.011	4.600±0.112	0.492±0.00	0.0000±0.00	1.768±0.020	10.66±0.105
10	3.184±0.09	0.0426±0.002	2.041±0.022	6.883±0.071	6.880±0.02	0.0519±0.005	0.909±0.028	3.245±0.138	0.409±0.01	0.0620±0.008	1.186±0.019	6.017±0.071
Mean	3.81±0.05	0.0107±0.001	0.46±0.008	4.70±0.042	3.43±0.07	0.0074±0.001	0.56±0.013	4.45±0.073	0.59±0.03	0.0102±0.002	0.79±0.020	7.20±0.099
Median	3.07	0.0070	0.26	3.70	1.51	0.0000	0.42	2.38	0.20	0.0000	0.75	5.81
Maximum	19.31	0.0426	2.04	21.80	11.97	0.0519	1.40	14.46	3.62	0.0620	1.77	18.58
Minimum	0.00	0.0000	0.00	0.00	0.00	0.0000	0.005	0.34	0.00	0.0000	0.02	2.48

Sample IDs 1-6 and 7-10 represent locations of Shibganj and Kahaloo, respectively.

Suppl. Table S6: Calculated bioconcentration factor (BCF) in various vegetables cultivated in Bogra district of Bangladesh

Sample	Radish				Brinjal				Country bean			
ID	Cr	Cd	Ni	Pb	Cr	Cd	Ni	Pb	Cr	Cd	Ni	Pb
1	2.84	0.53	0.00	0.03	3.45	0.52	0.34	0.00	3.22	0.06	0.24	0.00
2	3.40	0.14	0.33	0.00	4.85	0.64	0.22	0.00	1.57	0.00	0.26	0.00
3	6.11	0.74	0.03	0.62	3.40	5.75	0.00	0.25	2.71	0.06	0.00	0.33
4	0.55	0.00	0.04	0.00	1.17	0.00	0.00	0.49	2.25	0.00	0.00	0.05
5	0.48	0.00	0.19	0.18	2.23	0.21	0.00	0.29	3.53	0.00	0.24	0.42
6	1.13	0.00	0.01	0.91	2.18	0.25	0.20	0.49	0.00	0.00	0.17	0.50
7	0.00	0.55	0.36	0.24	5.24	0.83	0.32	0.17	3.57	0.16	0.20	0.15
8	1.61	0.00	0.30	0.02	0.00	0.36	0.24	0.01	0.64	0.00	0.29	0.30
9	3.74	0.00	0.30	0.40	5.43	0.00	0.20	0.46	4.79	0.00	0.11	0.46
10	2.13	0.59	0.44	0.51	0.24	0.64	0.19	0.54	2.65	0.37	0.25	0.50
Mean	3.22	0.26	0.20	0.29	4.78	0.92	0.17	0.27	2.49	0.07	0.18	0.27
Median	2.48	0.07	0.24	0.21	3.43	0.44	0.20	0.27	2.68	0.00	0.22	0.32
Maximum	6.11	0.74	0.44	0.91	5.43	5.75	0.34	0.54	4.79	0.37	0.29	0.50
Minimum	0.00	0.00	0.00	0.00	0.00	0.00	0.00	0.00	0.00	0.00	0.00	0.00

Sample	Onion				Chilli				Potato			
ID	Cr	Cd	Ni	Pb	Cr	Cd	Ni	Pb	Cr	Cd	Ni	Pb
1	8.95	0.19	0.01	0.07	3.47	0.18	0.27	0.02	0.29	0.26	0.22	0.16
2	4.52	0.68	0.17	0.04	1.62	0.00	0.13	0.03	0.34	0.00	0.17	0.14
3	2.03	0.28	0.00	0.00	4.77	0.00	0.01	0.08	1.32	0.00	0.01	0.45
4	4.08	0.22	0.12	0.35	4.21	0.00	0.01	0.05	0.00	0.00	0.14	0.14
5	3.53	0.06	0.10	0.31	0.65	0.00	0.07	0.03	0.00	0.00	0.16	0.26
6	0.00	0.00	0.19	1.82	0.00	0.00	0.29	0.49	0.00	0.00	0.25	0.46
7	0.28	0.50	0.03	0.37	0.48	0.17	0.00	0.81	0.00	0.31	0.14	0.96
8	0.71	0.00	0.31	0.00	0.00	0.00	0.32	0.52	0.00	0.00	0.16	0.29
9	3.83	0.00	0.12	0.47	0.75	0.00	0.09	0.25	0.33	0.00	0.46	0.52
10	4.09	0.91	0.68	0.59	5.62	0.70	0.19	0.18	0.31	0.77	0.23	0.30
Mean	3.51	0.28	0.17	0.40	2.16	0.11	0.14	0.25	0.26	0.13	0.19	0.37
Median	3.68	0.20	0.12	0.33	1.18	0.00	0.11	0.13	0.14	0.00	0.17	0.30
Maximum	8.95	0.91	0.68	1.82	5.62	0.70	0.32	0.81	1.32	0.77	0.46	0.96
Minimum	0.00	0.00	0.00	0.00	0.00	0.00	0.00	0.02	0.00	0.00	0.01	0.14

Sample IDs 1-6 and 7-10 represent locations of Shibganj and Kahaloo, respectively.

Suppl. Table S7: Calculated chronic daily intake (CDI) and hazard quotient (HQ) values of non-carcinogenic human health risks due to dietary intake of toxic metals from various vegetables cultivated in Bogra district of Bangladesh.

Name of vegetable	Location ID	CDI for an adult				HQ for an adult			
		Pb	Ni	Cd	Cr	Pb	Ni	Cd	Cr
Country bean	1	0.00E+00	9.09E-06	4.13E-08	9.16E-05	0.00000	0.000455	0.000041	0.09165
	2	0.00E+00	1.19E-05	0.00E+00	2.64E-05	0.00000	0.000594	0.000000	0.02639
	3	6.75E-05	0.00E+00	2.87E-08	7.65E-05	0.01876	0.000000	0.000029	0.07650
	4	8.84E-06	0.00E+00	0.00E+00	1.20E-05	0.00246	0.000000	0.000000	0.01203
	5	8.22E-05	8.02E-06	0.00E+00	5.23E-05	0.02284	0.000401	0.000000	0.05229
	6	1.06E-04	8.41E-06	0.00E+00	0.00E+00	0.02948	0.000421	0.000000	0.00000
	7	2.94E-05	6.86E-06	1.20E-07	3.06E-05	0.00816	0.000343	0.000120	0.03059
	8	5.95E-05	1.46E-05	0.00E+00	5.48E-06	0.01654	0.000730	0.000000	0.00548
	9	9.67E-05	4.40E-06	0.00E+00	7.39E-05	0.02686	0.000220	0.000000	0.07394
	10	1.03E-04	1.30E-05	3.09E-07	3.65E-05	0.02857	0.000648	0.000309	0.03646
Onion	1	9.54E-06	3.01E-07	9.30E-08	2.41E-04	0.00265	0.000015	0.000093	0.24138
	2	4.81E-06	5.36E-06	2.50E-07	5.37E-05	0.00134	0.000268	0.000250	0.05370
	3	0.00E+00	0.00E+00	9.09E-08	4.06E-05	0.00000	0.000000	0.000091	0.04058
	4	4.87E-05	2.91E-06	8.33E-08	1.54E-05	0.01352	0.000145	0.000083	0.01541
	5	4.37E-05	2.27E-06	2.50E-08	3.70E-05	0.01214	0.000114	0.000025	0.03703
	6	2.72E-04	6.61E-06	0.00E+00	0.00E+00	0.07568	0.000331	0.000000	0.00000
	7	5.28E-05	6.69E-07	2.66E-07	1.70E-06	0.01466	0.000033	0.000266	0.00170
	8	0.00E+00	1.10E-05	0.00E+00	4.32E-06	0.00000	0.000548	0.000000	0.00432
	9	7.00E-05	3.47E-06	0.00E+00	4.18E-05	0.01944	0.000174	0.000000	0.04184
	10	8.60E-05	2.55E-05	5.33E-07	3.98E-05	0.02390	0.001276	0.000533	0.03980
Chilli	1	4.01E-06	1.07E-05	1.27E-07	1.03E-04	0.00111	0.000537	0.000127	0.10252
	2	6.62E-06	6.22E-06	0.00E+00	2.82E-05	0.00184	0.000311	0.000000	0.02818
	3	1.77E-05	4.55E-07	0.00E+00	1.40E-04	0.00491	0.000023	0.000000	0.13965
	4	9.49E-06	1.89E-07	0.00E+00	2.33E-05	0.00264	0.000009	0.000000	0.02331
	5	6.60E-06	2.32E-06	0.00E+00	1.00E-05	0.00183	0.000116	0.000000	0.01003
	6	1.07E-04	1.46E-05	0.00E+00	0.00E+00	0.02984	0.000728	0.000000	0.00000
	7	1.69E-04	5.82E-08	1.37E-07	4.30E-06	0.04688	0.000003	0.000137	0.00430
	8	1.07E-04	1.63E-05	0.00E+00	0.00E+00	0.02962	0.000817	0.000000	0.00000
	9	5.37E-05	3.67E-06	0.00E+00	1.20E-05	0.01491	0.000184	0.000000	0.01197
	10	3.79E-05	1.06E-05	6.05E-07	8.03E-05	0.01052	0.000530	0.000605	0.08027
Potato	1	1.88E-03	5.28E-04	1.16E-05	5.25E-04	0.52328	0.026412	0.011595	0.52527
	2	1.65E-03	4.89E-04	0.00E+00	3.70E-04	0.45950	0.024431	0.000000	0.37007
	3	5.89E-03	1.49E-05	0.00E+00	2.42E-03	1.63735	0.000745	0.000000	2.41593
	4	1.81E-03	3.08E-04	0.00E+00	0.00E+00	0.50261	0.015380	0.000000	0.00000
	5	3.27E-03	3.59E-04	0.00E+00	0.00E+00	0.90796	0.017954	0.000000	0.00000
	6	6.24E-03	7.91E-04	0.00E+00	0.00E+00	1.73331	0.039530	0.000000	0.00000
	7	1.24E-02	3.14E-04	1.51E-05	0.00E+00	3.44144	0.015690	0.015133	0.00000
	8	3.74E-03	5.13E-04	0.00E+00	0.00E+00	1.03948	0.025650	0.000000	0.00000
	9	7.11E-03	1.18E-03	0.00E+00	3.28E-04	1.97391	0.058938	0.000000	0.32813
	10	4.01E-03	7.91E-04	4.13E-05	2.73E-04	1.11417	0.039530	0.041333	0.27267

Contd.

Name of vegetable	Location ID	CDI for an adult				HQ for an adult			
		**Pb**	**Ni**	**Cd**	**Cr**	**Pb**	**Ni**	**Cd**	**Cr**
Brinjal	1	0.00E+00	7.57E-05	2.13E-06	5.82E-04	0.00000	0.003786	0.002127	0.58202
	2	0.00E+00	5.82E-05	1.99E-06	4.83E-04	0.00000	0.002912	0.001992	0.48256
	3	3.06E-04	8.79E-07	1.55E-05	5.68E-04	0.08506	0.000044	0.015500	0.56811
	4	5.67E-04	0.00E+00	0.00E+00	3.70E-05	0.15741	0.000000	0.000000	0.03698
	5	3.35E-04	0.00E+00	7.47E-07	1.96E-04	0.09298	0.000000	0.000747	0.19584
	6	6.19E-04	5.78E-05	5.86E-07	1.40E-04	0.17200	0.002892	0.000586	0.14023
	7	2.03E-04	6.49E-05	3.71E-06	2.66E-04	0.05632	0.003244	0.003709	0.26584
	8	1.24E-05	6.92E-05	9.45E-07	0.00E+00	0.00345	0.003461	0.000945	0.00000
	9	5.77E-04	4.67E-05	0.00E+00	4.96E-04	0.16031	0.002333	0.000000	0.49620
	10	6.52E-04	5.82E-05	3.15E-06	1.92E-05	0.18117	0.002910	0.003145	0.01922
Radish	1	6.92E-06	0.00E+00	4.91E-07	1.10E-04	0.00192	0.000000	0.000491	0.10972
	2	0.00E+00	2.05E-05	1.02E-07	7.75E-05	0.00000	0.001025	0.000102	0.07749
	3	1.72E-04	1.21E-06	4.60E-07	2.34E-04	0.04772	0.000060	0.000460	0.23436
	4	0.00E+00	1.96E-06	0.00E+00	4.02E-06	0.00000	0.000098	0.000000	0.00402
	5	4.93E-05	8.54E-06	0.00E+00	9.70E-06	0.01371	0.000427	0.000000	0.00970
	6	2.61E-04	3.40E-07	0.00E+00	1.67E-05	0.07257	0.000017	0.000000	0.01672
	7	6.38E-05	1.69E-05	5.69E-07	0.00E+00	0.01773	0.000847	0.000569	0.00000
	8	6.65E-06	2.03E-05	0.00E+00	1.88E-05	0.00185	0.001017	0.000000	0.01876
	9	1.13E-04	1.59E-05	0.00E+00	7.84E-05	0.03141	0.000793	0.000000	0.07839
	10	1.42E-04	3.18E-05	6.68E-07	3.97E-05	0.03938	0.001588	0.000668	0.03974
	Mean	8.96E-04	1.01E-04	1.70E-06	1.43E-04	0.24882	0.005028	0.001697	0.14282
	Median	7.61E-05	1.07E-05	1.25E-08	3.70E-05	0.02114	0.000534	0.000013	0.03700
	Maximum	1.24E-02	1.18E-03	4.13E-05	2.42E-03	3.44144	0.058938	0.041333	2.41593
	Minimum	0.00E+00	0.00E+00	0.00E+00	0.00E+00	0.00000	0.000000	0.000000	0.00000

Sample IDs 1-6 and 7-10 represent locations of Shibganj and Kahaloo, respectively.

Suppl. Table S8: Calculated hazard index (HI) and incremental lifetime cancer risk (ILCR) values due to dietary intake of toxic metals from various vegetables cultivated in Bogra district of Bangladesh.

Name of vegetable	Location ID	Hazard index (HI) values for an adult	Calculated ILCR for an adult			Total ILCR (∑ILCR)
			Pb	Ni	Cd	
Country bean	1	0.09214	0.00E+00	8.28E-06	6.19E-07	8.89E-06
	2	0.02698	0.00E+00	1.08E-05	0.00E+00	1.08E-05
	3	0.09529	5.74E-07	0.00E+00	4.31E-07	1.01E-06
	4	0.01449	7.52E-08	0.00E+00	0.00E+00	7.52E-08
	5	0.07554	6.99E-07	7.30E-06	0.00E+00	8.00E-06
	6	0.02990	9.02E-07	7.66E-06	0.00E+00	8.56E-06
	7	0.03921	2.50E-07	6.25E-06	1.79E-06	8.29E-06
	8	0.02274	5.06E-07	1.33E-05	0.00E+00	1.38E-05
	9	0.10102	8.22E-07	4.00E-06	0.00E+00	4.83E-06
	10	0.06598	8.74E-07	1.18E-05	4.64E-06	1.73E-05
Onion	1	0.24414	8.11E-08	2.74E-07	1.40E-06	1.75E-06
	2	0.05556	4.09E-08	4.87E-06	3.75E-06	8.67E-06
	3	0.04067	0.00E+00	0.00E+00	1.36E-06	1.36E-06
	4	0.02916	4.14E-07	2.65E-06	1.25E-06	4.31E-06
	5	0.04931	3.72E-07	2.07E-06	3.75E-07	2.82E-06
	6	0.07601	2.32E-06	6.02E-06	0.00E+00	8.33E-06
	7	0.01666	4.49E-07	6.09E-07	3.99E-06	5.05E-06
	8	0.00486	0.00E+00	9.97E-06	0.00E+00	9.97E-06
	9	0.06145	5.95E-07	3.16E-06	0.00E+00	3.76E-06
	10	0.06551	7.31E-07	2.32E-05	7.99E-06	3.19E-05
Chilli	1	0.10430	3.41E-08	9.77E-06	1.91E-06	1.17E-05
	2	0.03033	5.63E-08	5.66E-06	0.00E+00	5.72E-06
	3	0.14459	1.50E-07	4.14E-07	0.00E+00	5.64E-07
	4	0.02596	8.07E-08	1.72E-07	0.00E+00	2.52E-07
	5	0.01198	5.61E-08	2.11E-06	0.00E+00	2.17E-06
	6	0.03057	9.13E-07	1.33E-05	0.00E+00	1.42E-05
	7	0.05131	1.43E-06	5.30E-08	2.05E-06	3.54E-06
	8	0.03043	9.06E-07	1.49E-05	0.00E+00	1.58E-05
	9	0.02706	4.56E-07	3.34E-06	0.00E+00	3.80E-06
	10	0.09192	3.22E-07	9.65E-06	9.07E-06	1.90E-05
Potato	1	1.08655	1.60E-05	4.81E-04	1.74E-04	6.71E-04
	2	0.85400	1.41E-05	4.45E-04	0.00E+00	4.59E-04
	3	4.05403	5.01E-05	1.36E-05	0.00E+00	6.37E-05
	4	0.51799	1.54E-05	2.80E-04	0.00E+00	2.95E-04
	5	0.92592	2.78E-05	3.27E-04	0.00E+00	3.55E-04
	6	1.77284	5.30E-05	7.19E-04	0.00E+00	7.72E-04
	7	3.47227	1.05E-04	2.86E-04	2.27E-04	6.18E-04
	8	1.06513	3.18E-05	4.67E-04	0.00E+00	4.99E-04
	9	2.36098	6.04E-05	1.07E-03	0.00E+00	1.13E-03
	10	1.46770	3.41E-05	7.19E-04	6.20E-04	1.37E-03

Contd.

Name of vegetable	Location ID	Hazard index (HI) values for an adult	Calculated ILCR for an adult			Total ILCR (∑ILCR)
			Pb	Ni	Cd	
Brinjal	1	0.58793	0.00E+00	6.89E-05	3.19E-05	1.01E-04
	2	0.48746	0.00E+00	5.30E-05	2.99E-05	8.29E-05
	3	0.66871	2.60E-06	8.00E-07	2.32E-04	2.36E-04
	4	0.19438	4.82E-06	0.00E+00	0.00E+00	4.82E-06
	5	0.28957	2.85E-06	0.00E+00	1.12E-05	1.41E-05
	6	0.31570	5.26E-06	5.26E-05	8.79E-06	6.67E-05
	7	0.32911	1.72E-06	5.90E-05	5.56E-05	1.16E-04
	8	0.00785	1.05E-07	6.30E-05	1.42E-05	7.73E-05
	9	0.65884	4.91E-06	4.25E-05	0.00E+00	4.74E-05
	10	0.20644	5.54E-06	5.30E-05	4.72E-05	1.06E-04
Radish	1	0.11213	5.88E-08	0.00E+00	7.36E-06	7.42E-06
	2	0.07862	0.00E+00	1.87E-05	1.52E-06	2.02E-05
	3	0.28260	1.46E-06	1.10E-06	6.90E-06	9.46E-06
	4	0.00412	0.00E+00	1.78E-06	0.00E+00	1.78E-06
	5	0.02383	4.19E-07	7.77E-06	0.00E+00	8.19E-06
	6	0.08930	2.22E-06	3.10E-07	0.00E+00	2.53E-06
	7	0.01914	5.42E-07	1.54E-05	8.53E-06	2.45E-05
	8	0.02162	5.65E-08	1.85E-05	0.00E+00	1.86E-05
	9	0.11059	9.61E-07	1.44E-05	0.00E+00	1.54E-05
	10	0.08138	1.21E-06	2.89E-05	1.00E-05	4.01E-05
Mean		0.39836	7.61E-06	9.15E-05	2.55E-05	1.25E-04
Median		0.08534	6.47E-07	9.71E-06	1.88E-07	1.28E-05
Maximum		4.05403	1.05E-04	1.07E-03	6.20E-04	1.37E-03
Minimum		0.00412	0.00E+00	0.00E+00	0.00E+00	7.52E-08

Sample IDs 1-6 and 7-10 represent locations of Shibganj and Kahaloo, respectively.
